# Synchronous Cation-Driven and Anion-Driven Polypyrrole-Based Yarns toward In-Air Linear Actuators

**DOI:** 10.1021/acs.chemmater.4c00873

**Published:** 2024-09-30

**Authors:** Amaia B. Ortega-Santos, Jose G. Martínez, Edwin W. H. Jager

**Affiliations:** Sensor and Actuator Systems, Department of Physics, Chemistry and Biology, Linköping University, 581 83 Linköping, Sweden

## Abstract

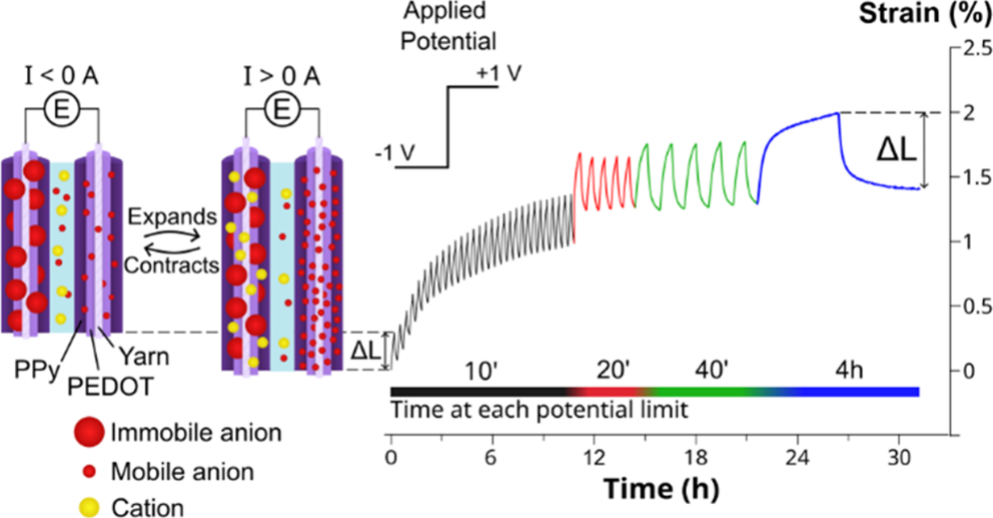

Conducting polymers (CP) have shown great features in building textile actuators. To date, most of the yarn-based or CP-yarn actuators have been operated in liquid electrolytes in a three-electrode-cell configuration, comprising an external counter and a reference electrode. For integration in textiles, a two-electrode system is needed, where both electrodes are in a yarn format. This can be achieved by having two CP-yarns, where one acts as the anode and the other as the cathode. For these two CP-yarns to operate synchronically, they both need to expand (or contract) during opposite reactions. This can be achieved by doping one CP-yarn with mobile anions that will expand during oxidation, while the other CP-yarn should be doped with immobile anions expanding during reduction. As a result, the same movement is created upon opposite redox reactions, both collaborating with the actuation in the same direction without the need for an external passive electrode to close the electrical circuit, which could oppose or hinder the movement. Most of the studies on textile actuators are based on cation-driven CP-yarn actuators, while little is known about anion-driven systems in CP-yarn actuators. Here, we first present a study of the effect of the dopants, solvents, and polymer layer combinations on the mechanism and strain of CP-yarns. The CP-yarns are coated with two layers: an inner poly(3,4-ethylenedioxythiophene) (PEDOT) layer and the outer and active polypyrrole (PPy) layer. According to our results, the dopant of the inner PEDOT layer seems to affect the actuation mechanism of the outer PPy layer and, thereby, of the whole CP-yarn actuator, influencing the direction of the movement and enhancing or hindering the total strain of the actuator. We show that a CP-yarn coated with PEDOT(Tos)/PPy(ClO_4_) and actuated in LiClO_4_ aqueous solution showed a pure anion-driven actuation. Next, based on the latter results, we demonstrate for the first time the dual actuation of two CP-yarns, doped with two different dopants, ClO_4_^–^ and DBS^–^, actuating simultaneously driven by opposite redox reactions and exhibiting an average of 0.5% of strain, an important step toward in-air actuating yarns.

## Introduction

Conducting polymers’ (CP) unique features have brought wide attention to scientists and engineers in the past years, leading to a diverse range of applications: from flexible displays^1^ to organic photovoltaics,^2^ organic transistors,^3^ drug delivery,^4^ tissue engineering,^5^ and CP actuators.^6^ CPs are organic semiconducting materials. They are typically synthesized by oxidative coupling of the monomer and simultaneous oxidation of the backbone that concurrently causes doping of the polymer.^7−9^ Their characteristic single/double C bonds in the backbone enable the electron movement along the backbone in their doped state.^10^

Focusing on CP actuators, their movement is driven by the volume variation caused by the exchange of solvated ions in an electrolyte upon electrical stimulus.^11^ When a current or potential is applied, electrons are removed from the polymer backbone, leaving the polymer positively charged. Anions enter the polymer matrix to compensate the charge, doping the material and keeping the electroneutrality.^12^ The latter has been confirmed to be the driving mechanism of the actuation of the conducting polymers from the works of Inganäs and Otero.^7,13−17^ Since then, this has been confirmed by others, with strain and charge measurements,^18−22^ as well as by means of atomic force microscopy (AFM) or electrochemical quartz crystal microbalance (EQCM) measurements.^23,24^

Most commonly, oxidation of p-doped CP gives, depending on the size of the ion included during the electrochemical synthesis, i.e., the doping anion or dopant, two different types of actuation mechanisms.^15^ Anion exchange dominates if the doping anions are small or mobile (eq 1). When a reduction potential is applied to the CP, electrons are inserted in the polymer modifying the material toward its neutral state. The size of the anions prevents them from being trapped inside the polymer and they exit the material to compensate the charge. As a result, the CP contracts upon reduction. When the oxidation potential is applied instead, the polymer backbone becomes positively charged and the anions available in the electrolyte will enter the CP leading again to charge neutrality and causing the material to expand during oxidation. For instance, actuators doped with small perchlorate (ClO_4_^–^),^25^ bis(trifluoromethane) sulfonamide (TFSI^–^),^26,27^ and hexafluorophosphate (PF_6_^–^)^28^ anions have shown to be anion-dominated systems. On the contrary, when big/immobile doping anions are confined inside the matrix of the CP, these cannot freely enter and exit the material to balance the charge caused by the applied redox potentials. Therefore, the exchange of cations becomes predominant (eq 2). In this case, the CP swells upon reduction when the cations enter the polymer matrix and shrinks during oxidation when these cations exit it. Dodecylbenzenesulfonate (DBS^–^) is a well-known big/immobile doping anion that has shown great actuation results when actuated in the same sodium dodecylbenzenesulfonate (NaDBS) aqueous electrolyte, or in other salt solutions, e.g., polypyrrole (PPy) free-standing films doped with DBS^–^ anions actuated with TFSI^–^ in propylene carbonate, aqueous, or mixtures of the last two showed from 11.4% up to 26% of strain in the very first cycles.^29−32^ Thus, by observing the direction of the movement (swelling/shrinking) during both oxidation and reduction, it is possible to determine which are the main ionic species exchanged between the CP and the electrolyte. This could involve either small anions, which follow reaction 1 and cause swelling during oxidation and shrinking during reduction, or cations, which follow reaction 2 and result in swelling during reduction and shrinking during oxidation
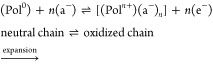
1
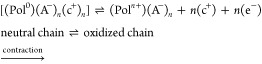
2Pol^0^ refers to the neutral polymer chain, whereas Pol^*n*+^ represents the oxidized state. a^–^ and A^–^ stand for the small/mobile and big/immobile anions, c^+^ for the cations, and e^–^ for the electrons. *n* refers to the number of molecules that take part in the reaction.

In practice, the system is far more complex. The presence of different species and the interaction between them, e.g., the dopant,^33^ the electrolyte of the actuation,^34^ the pH of the solution,^35,36^ the diverse type of bonding inside the polymer matrix, and the morphology of the polymer itself,^28^ or the effect of the electrochemical parameters, such as the applied potential window,^37^ and the scan rate,^20^ all influence and increase the complexity of the system.^38^ In fact, switched mechanisms or a combination of both anion and cation movements have also been reported.^39,40^ The effects of the solvent and the salt in the switching of the mechanism have been studied and yielded different results. It has been reported that the salt of the electrolyte determined the actuation mechanism.^32,41^ Furthermore, both groups showed that the direction of the movement gradually changed from cation to mixed and finally to a pure anion-driven system in the same solvent when prolonging the actuation. Other studies showed that PPy(DBS) films exchange cations when actuated in water or ethylene glycol and exchange anions when actuated in propylene carbonate or acetonitrile solutions.^42^ A similar effect has been observed in PPy(DBS) films actuated in LiClO_4_ propylene carbonate (anion-driven system) when substituting the solvent with acetonitrile (cation-driven system).^43^ The authors attributed the opposite mechanisms to the complexity of the interactions among ions, polymer, and solvent. It has also been shown that a considerable part of the volume change is caused by solvent molecules. The water molecules going in and out of the polymer follow the changes in the ionic content inside the polymer, referred to as osmotic pressure, which contributes significantly to the total actuation.^44,45^

Different arrangements of the conducting polymer in the actuator lead to different types of movements.^8,46^ For instance, free-standing polymer films shrink and expand linearly.^17^ Bending movement can be achieved by constructing bilayer and trilayer films by laminating the CP layer on one or two sides of a passive layer.^13^ Opposite sides shrink and swell, bending the films toward the extremes. Alternatively, by fixating the bilayer or trilayer films on two or more sides, the actuation direction changes resulting in a buckling movement.

The actuators presented in this work are conducting-polymer-based yarns (CP-yarns). These are regular, commercial yarns that were coated with two different conducting polymers. The first one, poly(3,4-ethylenedioxythiophene) (PEDOT), turns the yarn into a conductive substrate and enables subsequent electropolymerization of the second conducting polymer, polypyrrole (PPy). As a result, the CP-yarn expands and contracts under electrical stimuli in the axial direction, creating a linear movement.

Most of the CP actuators and in particular CP-yarns have been actuated in liquid electrolytes and very few in open air.^47−50^ In addition to the difficulty of transferring the electrolyte’s characteristics to air-stable ionogels, an in-air actuation introduces a new challenge: a two-electrode system is preferred for easier integration and control. In the case of linear actuators, ideally, the two electrodes should expand and contract simultaneously. In recent years, various configurations for in-air actuation have been proposed. For example, Plesse et al. presented a cylindrical, trilayer actuator. The first (inner) and the third (outer) layers were conducting polymers, whereas the second layer was an interpenetrated polymer network (IPN) that functioned as a solid-state electrolyte, in a coaxial arrangement. In such a configuration, the inner and outer layers are addressed as the working electrode and counter-reference electrode, respectively. The inner layer’s electrochemically induced movement was significantly greater than the movement of the counter electrode (CE), which was in the opposite direction. The difference between both strains led to a movement of the whole system in the directions of the dominating inner electrode.^48^ However, the opposing strain of the counter electrode movement resulted in a loss of efficiency in the system. The use of both anion- and cation-driven actuations in the two electrodes that comprise the actuator is another possible solution to build in-air linear actuators. The same group has recently presented a system that avoids the opposing movements using both the anion- and cation-driven actuation; by using two different polycationic and polyanionic ionogels, they forced carbon-nanotube-based yarns to exchange cations or anions with the gel, making them expand and contract simultaneously upon the opposite redox reactions.^51^ The latter do not need an external electrode to close the electrical circuit, and both counter and working electrodes contribute in the same direction to the movement of the actuator, enhancing the efficiency of the actuator.

**Figure 1:**
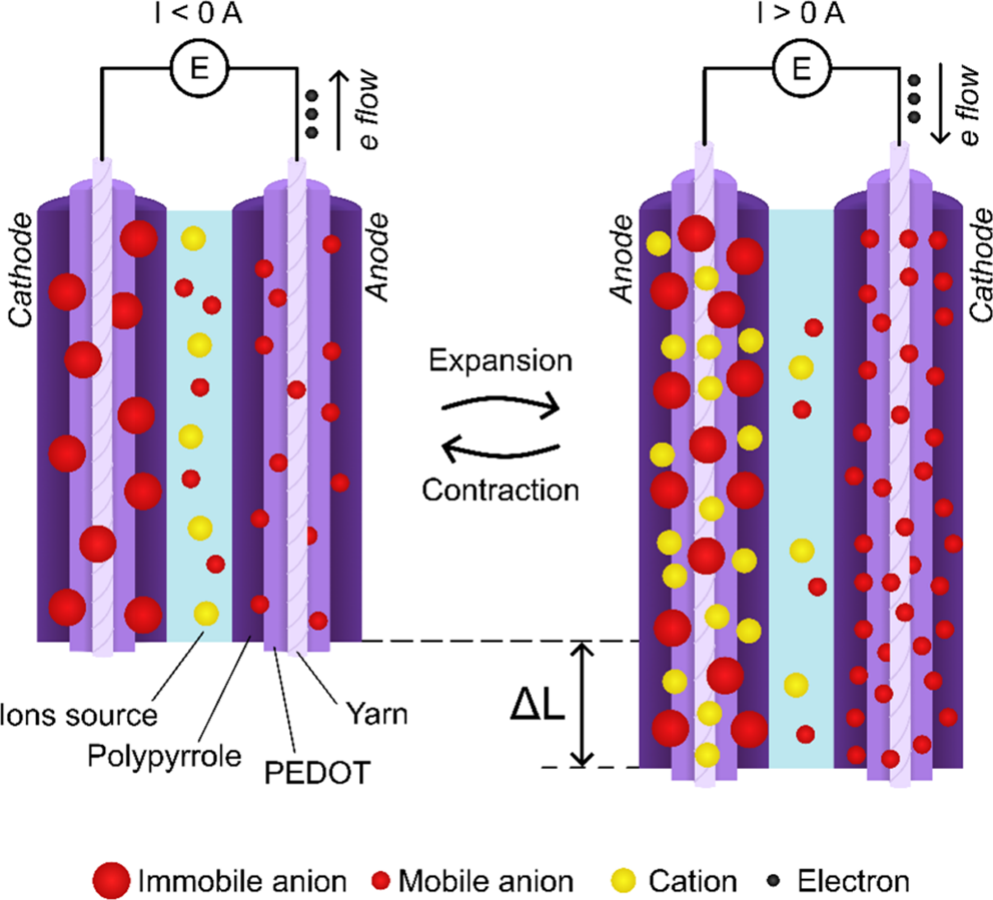
Schematic description of the simultaneous performance of two CP-yarns doped with small and big anions when subjected to alternating potentials.

Instead of using two different electrolytes or ionogels, the mechanism of the conducting polymer itself can be tuned by using different anion sizes during the electropolymerization as previously mentioned. For CP-yarns, this means that the yarn doped with small/mobile anions will expand upon oxidation by exchanging anions with the media (anion-driven), whereas the yarn doped with big/immobile anions will also expand but upon reduction by exchanging cations with the media (cation-driven) (Figure 1). When the current is reverted, the anion-driven yarn will shrink during reduction, and the cation-driven yarn will shrink upon oxidation. That is, movement in the same direction is triggered, while opposite redox reactions occur. The movement of both yarns in not hampering each other as in the case of ref (40) but augmenting each other, making the most of the total strain of the CP-yarns.

To construct such an in-air system based on the combination of two CP-yarns that expand and contract simultaneously, we need to find a pair of CP-yarns, doped with small and big anions, that will actuate in the same electrolyte with opposite mechanisms. While numerous studies focus on the anion-driven actuation of polypyrrole films electrogenerated on highly conducting metallic substrates, the majority of the textile-based actuators that have been reported were doped and moved in aqueous solutions prepared with NaDBS salt, resulting in pure cation-driven movement,^52,53^ showing strains up to 1.7% for single CP-yarns, and up to 3% for knitted fabrics.^54,55^ In these studies, we mainly focused on enhancing the strain of the CP-yarns by studying the impact of the core yarn in the actuation or by weaving and knitting the CP-yarns into textiles. Following the first track, it was found that by selecting soft and stretchable commercial yarns, the conducting polymer coating becomes the limiting factor in the actuation.^53^ It would also be possible to enhance the strain by weaving and knitting the yarns, as it was shown that these coated yarns, as the one selected for this work, could be used in woven and knitted fabrics.^55,56^ However, little is known about how changes in the electrolyte–polymer system affect the actuation of CP-yarns, a complex system with two different conducting polymer layers. Furthermore, to the best of our knowledge, no one has reported a pure anion-driven system in CP-yarn or textile actuators and therefore neither a two-electrode system based on a pair of CP-yarns with opposing actuation mechanisms.

In the first part of this paper, while seeking the anion-driven CP-yarn system, we present a study on the effect of dopants, solvents, and PPyand PEDOT layers on the actuation mechanism and strain of the CP-yarns. Based on these results, we then demonstrate the dual-actuation system with two yarns, one doped with ClO_4_^–^ small anions and the other one doped with DBS^–^ big anions, that actuate simultaneously in a two-electrode system and in a LiClO_4_ aqueous electrolyte. This study further sheds light on the complex interaction of multiple layers of conducting polymers and electrolytes. This will help the development of linear (textile) actuators by better understanding the effect of the electrolyte, solvent, and polymers on final actuation. By understanding the influence of these parameters, one will be able to build and tune more sophisticated actuators.

Second, the paper demonstrates the possibility of the dual actuation of two yarns based on the two actuation mechanisms. This is the first step toward the development of in-air dual-actuating CP-yarn systems, which could be further integrated into smart textiles.

## Experimental Section

### Materials

Nonconductive Viscose Rotor Spun Ne 30/1 yarns were provided by the Swedish School of Textiles (Borås University) and manufactured by Annapoorna Cotspin Co., India. Poly(3,4,-ethylenedioxythiophene) polystyrenesulfonate (PEDOT:PSS) (Heraeus), polyethylene glycol 400 (PEG400) (KEBO), themonomer liquid 3,4-ethylenedioxythiophene (EDOT) (Tokyo Chemical Industry Co.), and iron(III) *p*-toluenesulfonate [Fe(Tos)_3_] in butanol solution (Heraeus) were stored at 6 °C and used without further purification. Pyrrol (Sigma-Aldrich) monomer liquid was distilled under vacuum before use and stored at −20 °C. Sodium dodecylbenzenesulfonate (NaDBS), sodium trifluoromethanesulfonate (NaOTf), lithium bis(trifluoromethane) sulfonamide (LiTFSI), tetrabutylammonium trifluoromethanesulfonate (TBAOTf), 1-ethyl-3-methylimidazolium trifluoromethanesulfonate (EMImOTf), tetrabutylammonium hexafluorophosphate (TBAPF_6_), lithium perchlorate (LiClO_4_), and acetonitrile, all purchased from Sigma-Aldrich, were stored at room temperature, and employed without any previous treatments. All of the aqueous solutions were prepared with Milli-Q deionized water.

### Coating

**Figure 2:**
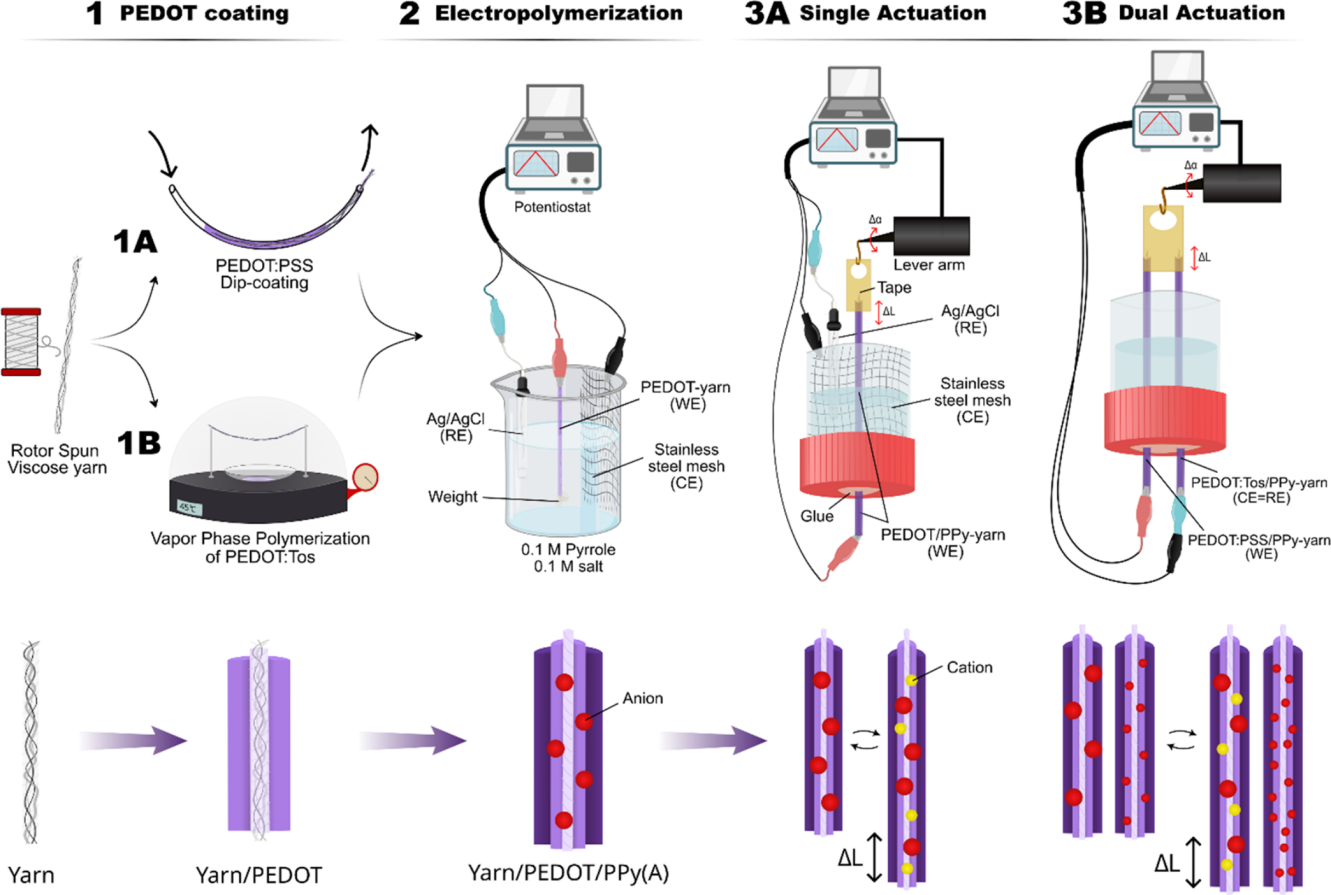
Schematic representation of the three main steps of the preparation and actuation of the yarns. Below: different polymer layers of the actuator.

The nonconductive viscose yarns were either coated with poly(3,4-ethylenedioxythiophene), polystyrenesulfonate, and polyethylene glycol mixture (PEDOT:PSS/PEG400) or poly(3,4-ethylenedioxythiophene) doped with tosylate (PEDOT(Tos)). In the first case, 35 cm viscose yarns were dip-coated by passing them seven times through a tube filled with a solution of 91.4% (v/v %) PEDOT:PSS and 8.6% PEG400 (Figure 2, step 1A). The 35-cm-long yarn was hung and left for drying between each coating. The PEDOT:PSS linear density was calculated by measuring the weight difference of the yarn before and after the dip-coating process and then dividing this by the length of the whole yarn, which was typically between 30 and 35 cm. The objective was to not exceed a maximum of 220 Ω/cm resistance; therefore, if needed, the yarn was dip-coated again until a lower value of resistance was obtained. The yarn was discarded if the optimal conductivity was not reached and if its linear density excedeed 300 μg/cm. From each long yarn, samples of 6–7 cm-long yarns were cut. The 6–7 cm-long PEDOT:PSS/PEG400-coated yarns’ resistance was kept below 1500 Ω by measuring it with a MultimetrixR DMM220 electronic multimeter. In the second case, bare yarns were first dip-coated with one layer by passing them once through a tube filled with a solution of iron(III) *p*-toluenesulfonate [Fe(Tos)_3_] in butanol solution. Next, yarns were exposed to 3,4-ethylenedioxythiophene (EDOT) monomer by using the vapor phase polymerization (VPP) method (Figure 2, step 1B) in a vacuum chamber for 20 min at 45 °C, 70 cmHg. In this case, the VPP coating was repeated only once. Again, 6–7-cm-long samples were cut from the long yarn. The linear resistance of the small PEDOT(Tos)-yarns was measured using the same multimeter.

### Electropolymerization

Polypyrrole was galvanostatically deposited from a pyrrole monomer solution in the presence of different anions in a three-electrode electrochemical cell on the PEDOT:PSS or PEDOT(Tos) layer by applying 0.5 mA for 10 000, 20 000, or 30 000 s. NaDBS, TBAOTF, NaOTf, EMImOTf LiTFSI, TBAPF_6_, and LiClO_4_ were used to polymerize the CP-yarns in either aqueous or acetonitrile solutions. The concentrations of the pyrrole and the salt of the electrolyte were kept constant at 0.1 M throughout all of the experiments. The electroactivity of the CP-yarn was corroborated by performing cyclic voltammetric scans before and after the polymerization between [−1, 0.3] V, vs Ag/AgCl (3 M NaCl) reference electrode (RE) (MF-2052 model from BASi Research Products) at 10 mV/s, for three cycles each, at room temperature. The limits of cyclic voltammetry were kept constant throughout all of the experiments unless otherwise mentioned. The CP-yarn (working electrode, WE) was clamped from an alligator clip, and the lower 4 cm of the sample was submerged in a 2.8 cm diameter tube filled with 45 mL of electrolyte solution (Figure 2, step 2). A nylon nonconductive screw (1 g) was clipped to the lower tip of the CP-yarn to keep it straight inside the liquid. A stainless steel mesh was used as the counter electrode (CE), which was immersed and attached to the walls of the tube, surrounding the CP-yarn. The control and measurements of the electrochemical parameters of the cell were performed with the Ivium Compactstat potentiostat combined with Iviumsoft software, both from Ivium Technologies. Each experiment was repeated three times.

### Actuation

The actuation of single CP-yarns was carried out by performing an isotonic (12.5 mN) chronoamperometry between [−1, 0.3] V vs Ag/AgCl (3 M NaCl) reference electrode for 10–15 cycles at 0.833 mHz (20 min period, 10 min at each potential limit), at room temperature. TBAOTf, NaOTf, and EMImOTf in acetonitrile and LiClO_4_ in aqueous solutions were used to actuate the CP-yarns. The salt concentration was kept constant and equal to 0.1 M throughout all of the experiments. The upper tip of the CP-yarn (WE) was hooked to the lever arm (Series 300B Lever System from Cambridge Technology, Inc.) with a Kapton tape. The lower tip was glued (Power Epoxi from Loctite) to a bottom lid of a 2.8 cm diameter and 3 cm high cylindrical tube and connected to the WE (Figures 2, step 3A). The stainless steel counter electrode mesh was immersed in the solution and attached to the cell walls, whereas the Ag/AgCl (3 M NaCl) reference electrode was inserted by using a metallic clamp.

**Figure 3:**
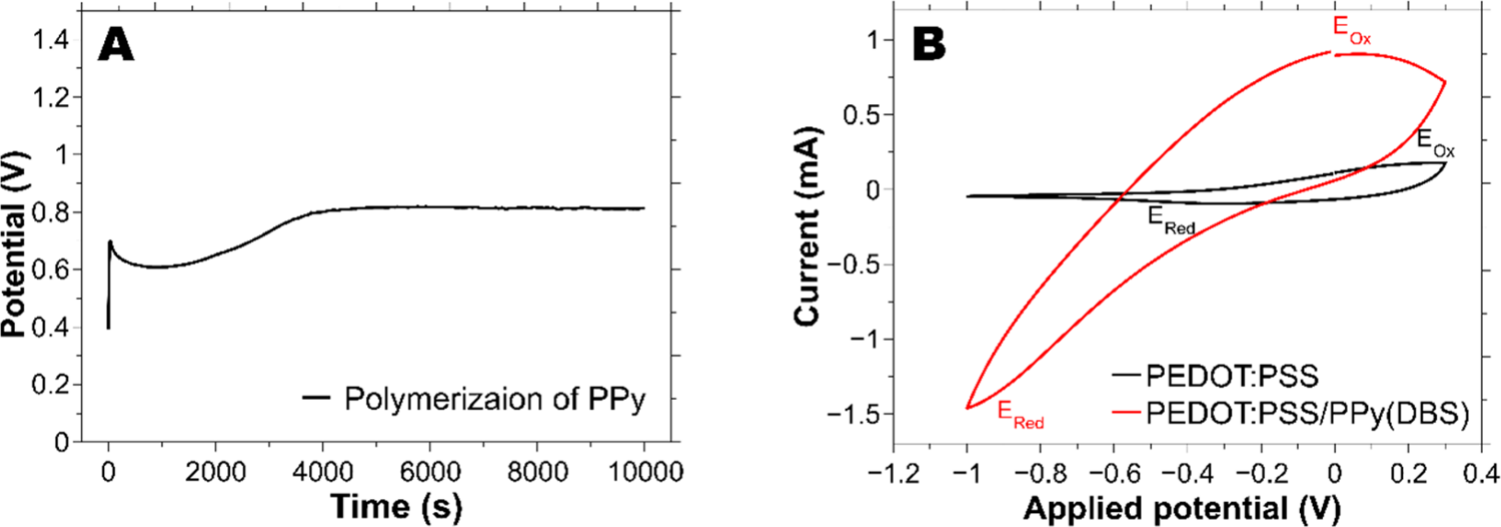
(A) Electropolymerization of PPy(DBS) onto the PEDOT:PSS in 0.1 M NaDBS and 0.1 M pyrrole aqueous solution. Current was set to 0.5 mA for 10 000 s. (B) Cyclic voltammetry of PEDOT:PSS and PPy(DBS) between [−1, 0.3] V at 10 mV/s in 0.1 M pyrrole and 0.1 M NaDBS aqueous solution.

The actuation of the dual CP-yarn actuator was carried out by performing an isotonic chronoamperometry between [−1, 1] V for a 20 min period, 10 min at each potential limit, until stable contraction and expansion were observed. Then, time was increased to 20 min (five cycles), 40 min (five cycles), and finally up to 2 h at each potential limit (one cycle), where the actuation reached a quasi-plateau value. 0.1 M LiClO_4_ in aqueous solution was used to actuate the two CP-yarns. The two CP-yarns were kept parallel to each other and 1 cm apart. They were hooked to the lever arm with the same Kapton tape. The lower tips were glued (Power Epoxi from Loctite) to the bottom lid of a 2.8 cm diameter and 3 cm high cylindrical tube (see Figure 2, step 3B). The cation-driven yarn was set as the working electrode, and the anion-driven CP-yarn was connected to the counter electrode and shortcut to the reference electrode, making it a two-electrode system.

The elongation of the yarn was measured using a lever arm (Figure 2, steps 3A and 3B). The yarn was connected to the lever arm with a metallic arm that was hooked perpendicular to it. It can operate in two modes: isotonic and isometric. For both single-actuation and dual-actuation experiments, the lever arm was configured in isotonic mode: a bias force was set to 12.5 mN, which was kept constant throughout all the experiments, while the elongation of the yarn was measured. When the yarn expands and contracts linearly (up and down), the lever arm rotates at an equivalent angle. The lever arm transduces this rotation into an output signal in volts, which is related to the movement of the yarn as 1 V per 500 μm, with a length resolution of 1 μm. The elongation-equivalent electrical signal from the lever arm (1 V ∼ 0.5 mm), together with the current and charge versus time, was recorded by the Autolab PGSTAT204 potentiostat from Metrohm Autolab combined with the NOVA 2.1.4 software. Finally, the linear strain was calculated according to eq 3
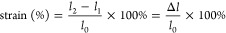
3where *l*_1_ and *l*_2_ correspond to the maximum and minimum strain values in each cycle, *l*_0_ refers to the initial length of the CP-yarn immersed in the solution, which was 2 cm. Each actuation experiment was repeated three times with three different yarns. All of the standard deviations were calculated from the three different experiments.

## Results and Discussion

### Effect of the Actuation Electrolyte

To study the effect of the ions in the actuation electrolyte, all of the CP-yarns were first dip-coated in PEDOT:PSS–PEG400 and electropolymerized in 0.1 M pyrrole and 0.1 M NaDBS aqueous solution. Since all of the CP-yarns were electropolymerized under the same electrochemical parameters and doped with the same large DBS^–^ anion, they showed similar chronopotentiograms and electrochemical activity.

Figure 3A shows the typical potential response of the galvanostatic electropolymerization of PPy(DBS) in aqueous solution. Because of the sudden current application, the graph presents a potential step in the first few seconds. Then, it drops to a minimum value, and after few minutes it starts to increase again until it reaches a plateau value at ∼0.79 V. A similar behavior has been shown in different studies, although the stabilization of the potential is quicker and the valley is shallower than the one shown in this work.^26,57,58^ These minor dissimilarities are attributed to different surfaces of the samples. The latter studies used flat metallic surfaces, whereas our coated CP-yarns have a heterogeneous and less conductive fibrous outer surface. Hence, the polymerization is reduced, delaying the stabilization. In addition, the underneath PEDOT layer may also consume charge for its own oxidation, which would reduce the electropolymerization rate during such oxidation.^59^ A typical PPy(DBS) cyclic voltammetry graph is shown in Figure 3B (red line), which is compared to prior electroactivity of the CP-yarn coated with only PEDOT:PSS (black line). The higher current for the PPy-coated yarns indicates successful PPy deposition. The oxidation and reduction peaks, *E*_ox_ and *E*_red_, of both PEDOT:PSS and PEDOT:PSS/PPy(DBS) are distinguished and at their expected positions when cycled between [−1, 0.3] V at 10 mV/s.^59^

**Figure 4:**
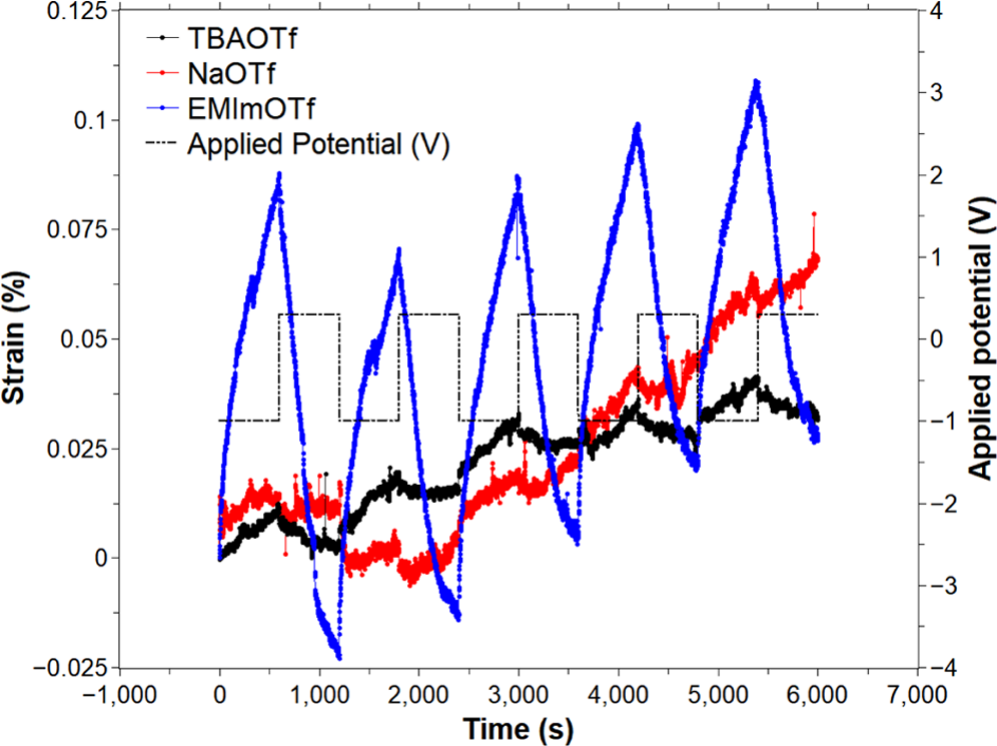
Last five cycles of PEDOT:PSS/PPy(DBS)-yarns’ actuation. [−1, 0.3] V square wave potential was applied at 0.83 mHz in 0.1 M NaOTf, TBAOTf, and EMImOTf acetonitrile solution.

Next, CP-yarns doped with DBS^–^ immobile anions were actuated in three different electrolytes: 0.1 M TBAOTf, NaOTf, and EMImOTf acetonitrile solutions. Note that the anion of the salt was always the same (OTf^–^) and was compatible with ionic gels, enabling the possibility of taking the yarns from the liquid systems to the in-air configurations.^60^Figure 4 shows the last 5 out of 15 cycles of the strain response of the CP-yarns in the three different electrolytes. The cycle was set to the [−1 and 0.3] V square wave potential at 0.83 mHz. CP-yarns actuated in EMImOTf presented an average of 0.1 ± 0.01 and 0.04 ± 0.04% expansion and contraction strains, followed by TBAOTf with 0.02 ± 0.002 and 0.01 ± 0.01% and last, NaOTf with 0.01 ± 0.003 and 0.01 ± 0.01% (see Table 1). All of these strain values are lower than those obtained when DBS^–^-doped PPy-yarns are actuated in NaDBS aqueous solution.^54^ Note that there are differences in the type of the core yarns (viscose ring rotor 1 ply yarns vs viscose rotor spun Ne 30/1), and the actuation solvent (aqueous vs acetonitrile) and salts (NaDBS vs EMImOTf, TBAOTf, and NaOTf) used in ref (55) which can lead to different results and will be discussed in the following sections. Also, all actuations shown in Figure 4, and in most of the actuation figures in this paper, exhibit a positive slope. We believe that this can be caused by the mechanical relaxation effects, creeping effects, and some irreversible reduction of the polymer,^61,62^ an effect that is more noticeable in the first actuation cycles, as shown in Figure S1 and Table S1, where the full actuations of these yarns are displayed. Last, the actuation mechanisms were cation-driven, expanding during reduction and contracting during oxidation, as expected from a DBS-doped, PPy-coated CP-yarn.

**Table 1:** Effect of the Actuation Electrolyte on the Strain and Mechanism of CP-Yarns Doped with Large/Immobile DBS Anions^a^

combinations								
				actuation				
coating	polymerization of PPy					average strain		
polymer	time (s)	dopant	solvent	electrolyte	solvent	reduction (%)	oxidation (%)	mechanism
PEDOT:PSS	10 000	DBS^–^	AQ	TBAOTf	AN	0.02 ± 0.002	0.01 ± 0.01	cation-driven
PEDOT:PSS	10 000	DBS^–^	AQ	NaOTf	AN	0.01 ± 0.003	0.01 ± 0.01	cation-driven
PEDOT:PSS	10 000	DBS^–^	AQ	EMImOTf	AN	0.1 ± 0.01	0.044 ± 0.04	cation-driven

a AN and AQ stand for acetonitrile and aqueous solutions. Errors in the average strain are calculated as the standard deviation of three actuations of three different samples.

Similar electropolymerization conditions suggest that the differences in the actuation electrolytes caused the variations in the strain values. Several authors agree that the shape and molecule size of the cations play an essential role in the swelling and shrinking capacity of the polymer.^33,42,43^ On the other hand, distinct electrolytes also induce different interactions with both the solvent and the polymer matrix. Throughout the whole redox process, electrolyte, solvent, and polymers finely interplay.^43^ Interactions such as polymer–polymer, polymer–solvent, polymer–anion, polymer–cation, solvent–anion, solvent–cation, and anion–cation arise in the system. By changing only the cation of the actuation, we influence three of those interactions. Therefore, it is difficult to say which pair-forming is enhancing or decreasing the actuation.

Overall, CP-yarns actuated in EMImOTf showed the highest strains. Hence, it is the selectedactuation salt used in the following experiments and potentially for the dual-actuation proof of concept. EMImOTf in the ionic liquid state is also a good candidate for the preparation of ionogels and, therefore, for actuators operating in-air.^60^ In the following section, CP-yarns are doped with small anions and actuated in EMImOTf acetonitrile solutions, and their strain and actuation mechanisms are investigated.

### Effect of the Dopant

To obtain the dual linear actuation described in Figure 1, it is necessary to have a second CP-coated yarn that presents a similar but anion-driven linear strain. CP-coated yarns were doped with three different small anions that have been shown to be mobile: TFSI^–^,^26,27^ OTf^–^,^33^ and PF_6_^–^,^28^ and the same 0.1 M EMImOTf acetonitrile solution was employed as the electrolyte for all of the actuations. As seen in Figure S1, regardless of the dopant, all of the chronopotentiograms of the electropolymerization show a similar response: an initial peak of the synthesis potential, followed by a reduction and a subsequent increase of the potential until it reached a steady-state value of 1.15 ± 0.10 V.

**Figure 5:**
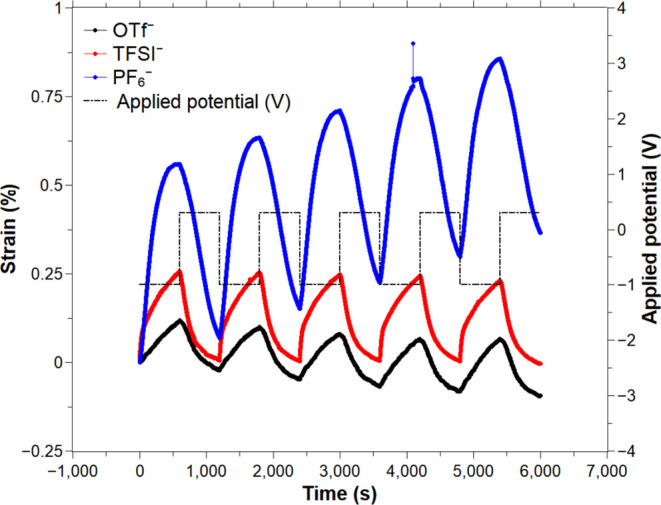
PPy(TFSI)-, PPy(OTf)-, and PPy(PF_6_)-yarns actuation in the last five cycles. Square wave potential was applied between [−1 and 0.3] V against Ag/AgCl (3 M NaCl) at 0.83 mHz in 0.1 M EMImOTf acetonitrile solution.

Figure 5 shows the last 5 of 15 cycles of the actuation of the CP-yarns doped with TFSI^–^, OTf^–^, and PF_6_^–^. The cycle was set to [−1, 0.3] V square wave potential at 0.83 mHz. Table 2 presents the average strain of the three combinations. Contrary to all other experiments in this work, PEDOT:PSS/PPy(TFSI)-yarn and PEDOT:PSS/PPy(OTf)-yarn showed a negative slope in the last 5 cycles, possibly due to some degradation after the first 10 cycles, which can also be seen in the full actuation graph in Figure S3, as well as in Table S1.

**Table 2:** Comparison of the Effect of Small/Mobile Dopants on the Strain and the Actuation Mechanism of CP-Yarns^a^

combinations								
				actuation				
coating	polymerization of PPy					average strain		
polymer	time (s)	dopant	solvent	electrolyte	solvent	reduction (%)	oxidation (%)	mechanism
PEDOT:PSS	10 000	OTf^–^	AN	EMImOTf	AN	0.06 ± 0.06	0.06 ± 0.08	cation-driven
PEDOT:PSS	10 000	TFSI^–^	AN	EMImOTf	AN	0.15 ± 0.08	0.14 ± 0.08	cation-driven
PEDOT:PSS	10 000	PF_6_^–^	AN	EMImOTf	AN	0.34 ± 0.19	0.29 ± 0.15	cation-driven

a Solv represents solvent. AN stands for acetonitrile solvent. Errors in the average strain are calculated as the standard deviation of three actuations of three different samples.

PPy(PF_6_)-yarns actuated in 0.1 M EMImOTf in acetonitrile present the largest average strain, 0.34 ± 0.19% when expanding and 0.29 ± 0.15% when contracting. Yarns coated with PPy doped with TFSI^–^ anions actuated at an average of 0.15 ± 0.08% during expansion and 0.14 ± 0.08% during contraction. PPy(OTf)-coated yarns showed the lowest strain (0.06 ± 0.06%) for both contraction and expansion. Contrary to the literature, all of these CP-yarns exhibited the cation-driven mechanism, expanding during reduction and contracting during oxidation, rather than the expected anion-driven mechanism, in which the polymer would expand during oxidation and contract during reduction.^27,28,33^ This indicates that the actuation is mainly driven by the exchange of EMIm^+^ cations rather than by the movement of OTf^–^, TFSI^–^, or PF_6_^–^ anionic dopants. Therefore, such CP-yarns cannot be used as the anion-driven yarn in a dual-actuation system since they would oppose the movement direction of the other yarn. In the following sections, we investigate the reasons why the mechanism is altered: first, by studying the combination of aqueous and acetonitrile solvents, and second, by studying the influence of the first PEDOT-coating layer in the mechanism.

### Effect of the Solvent

**Figure 6:**
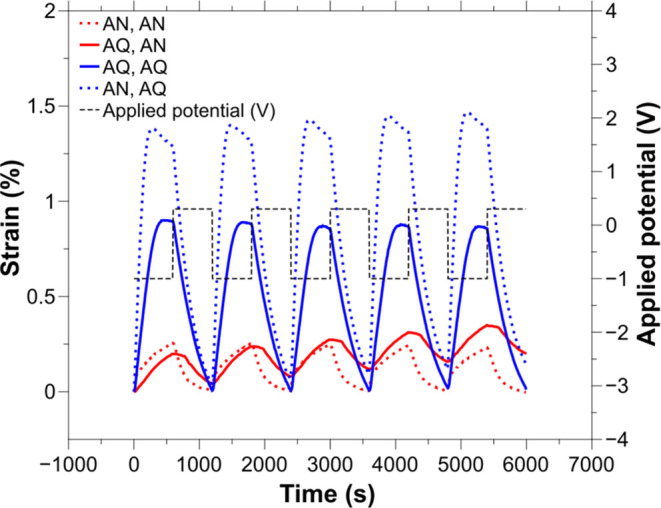
Effect of the solvent in the actuation response of PEDOT:PSS/PPy(TFSI)-yarns doped in acetonitrile (AN) or water (AQ) solutions and actuated in 0.1 M EMImOTf acetonitrile or water mixtures. [−1, 0.3] V square wave potentials were applied at 0.83 mHz.

The literature showed that in some cases, the nature of the solvent may influence the whole system and affect the mechanism of the CP-actuator. It has been speculated that the polarity and conductivity of the liquid play a role in the solubility and ability of the solvent to break the anion–cation bonds, which can eventually lead to the switch of the actuation mechanism.^34,42,63^ The number of solvent molecules that enter the matrix, driven by osmotic pressures or solvated with the ions, differs from one solvent to the other, which can also affect the actuation.^46,64−66^ Here, we investigate the effect of the solvent on CP-yarns’ strain and mechanism. Although CP-yarns coated with PPy(PF_6_) exhibited the greatest strain, the TBAPF_6_ is not water-soluble, so the system could not be tested in both aqueous and acetonitrile solutions. Therefore, CP-yarns doped with TFSI^–^ synthesized in both aqueous and acetonitrile solvents were actuated in 0.1 M EMImOTf aqueous and acetonitrile solutions (Figure 6 for the last five cycles, and Figure S4 for the full actuation). By keeping the salts in the polymerization and in the actuation the same, we investigated only the effect of the solvent. As seen in Figure 6, the CP-yarns polymerized in acetonitrile and actuated in water (blue dashed line) showed the greatest actuation. The actuation was driven by cations in the first half of the cathodic actuation (reduction), where the CP-yarn expands. The expansion actuation was reverted in the middle of the same cathodic cycle and switched to a more subtle anion-dominated actuation, where the CP-yarn contracted instead of expanded. No change in the actuation mechanism is perceived when anodic potentials are applied: the CP-yarn contracted during the whole duration of the oxidation, i.e., cation motion. The CP-yarns electropolymerized and actuated in water (blue solid line) showed smaller strain values and a less visible but still perceptible anion-driven mechanism also at the end of the reduction cycles. Again, the oxidation cycles were purely cation-driven. Last, the CP-yarns actuated in acetonitrile (red lines), regardless of the solvent used during the electropolymerization, showed a pure cation-driven and a remarkable smaller actuation. All yarns showed a cation-dominated actuation, but only on those yarns actuated in aqueous solutions could be witnessed a very slow anion-dominated actuation at the end of the cycle.

While the solvent did not exceedingly affect the mechanism in these CP-yarns, it can be decisive for enhancing their strain.^28,67^ CP-yarns actuated in aqueous mixtures displayed the largest strain. On average, the linear deformation in an aqueous electrolyte was 0.51 ± 0.32% when polymerized in an aqueous solution and 0.94 ± 0.36% when polymerized in acetonitrile (Table 3, rows 2 and 4). These values are lower than the strain obtained for the pure PPy films peeled off from the substrate, but close to the maximum actuation reported on CP-yarns.^54^ These results suggest that using acetonitrile in the actuation decreases the strain, whereas the aqueous solution enhances it, regardless of the solvent used during the electropolymerization. Yet, the solvent does not fully change the actuation mechanism: the mechanism is purely cation-driven for acetonitrile mixtures during the actuation and cation-dominated for the first half and slightly anion-dominated in the second half of the actuation for aqueous solutions.

**Table 3:** Comparison of the Effect of the Solvent on the Strain and Mechanism of CP-Yarns^a^

combinations								
				actuation				
coating	polymerization of PPy					average strain		
polymer	time (s)	dopant	solvent	electrolyte	solvent	reduction (%)	oxidation (%)	mechanism
PEDOT:PSS	10 000	TFSI^–^	AN	EMImOTf	AN	0.15 ± 0.08	0.14 ± 0.08	cation-driven
PEDOT:PSS	10 000	TFSI^–^	AQ	EMImOTf	AQ	0.51 ± 0.32	0.51 ± 0.31	cation-driven
PEDOT:PSS	10 000	TFSI^–^	AQ	EMImOTf	AN	0.11 ± 0.08	0.08 ± 0.06	cation-driven
PEDOT:PSS	10 000	TFSI^–^	AN	EMImOTf	AQ	0.94 ± 0.36	0.87 ± 0.33	cation-driven

a Solv represents solvent. AN and AQ stand for acetonitrile and aqueous solutions. Errors in the average strain are calculated as the standard deviation of three actuations of three different samples.

### Well-Known Anion-Driven Actuation

To further shed light on the switching from the anion- to cation-driven actuation mechanism, we used a well-known small mobile anion, ClO_4_^–^, as the dopant of PPy.^68^ This anion has shown to lead to anion-driven actuations in various systems.^53−55^ We fabricated PPy(ClO_4_)-yarns from an aqueous LiClO_4_ solution and actuated them in the same but monomer-free solution. Likewise, the actuation mechanism of ClO_4_^–^ anion has also been shown to be sensitive to the solvent; the results showed that the PPy(ClO_4_) actuation mechanism changed from anion- to cation-driven by replacing propylene carbonate with acetonitrile.^43^ Therefore, to ensure that the solvent will not affect the deformation direction, both polymerization and actuation were performed in aqueous solutions.

**Figure 7:**
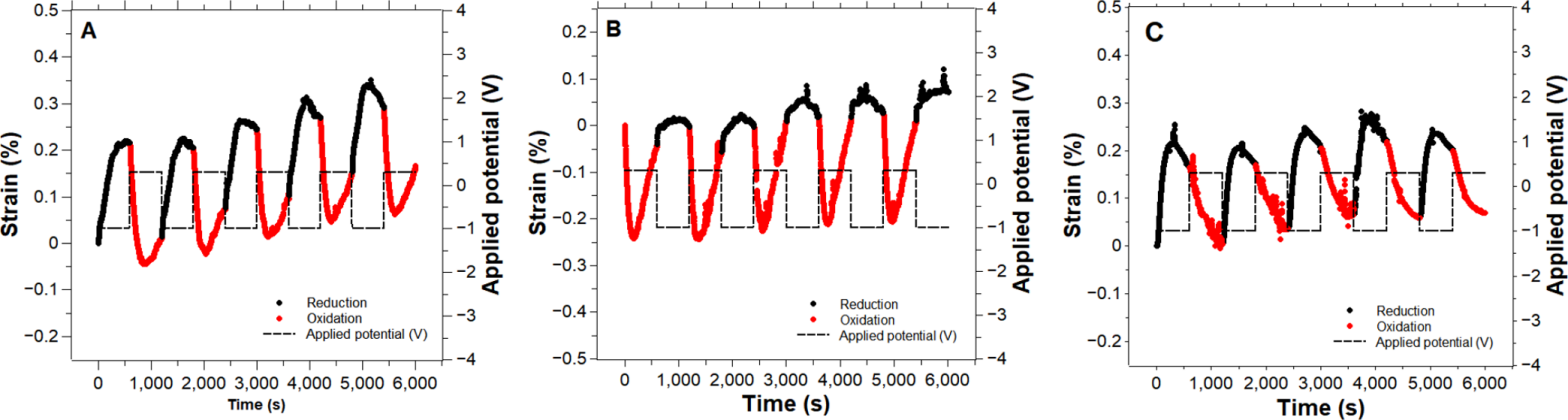
Actuation of PEDOT:PSS/PPy(ClO_4_) yarns actuated in 0.1 M aqueous LiClO_4_. [−1, 0.3] V square wave potentials were applied at 0.83 mHz for 15 cycles. Each graph represents one out of the three repetitions that were performed.

Figure 7 shows the last cycles of the actuation of three repetitions of PPy(ClO_4_)-yarns electropolymerized for 10 000 s, and Figure S5 displays the full actuation of these samples. Both anion- and cation-driven actuations could be distinguished at both anodic and cathodic potentials. For instance, Figure 7A shows how the CP-yarn contracted and thereafter started to expand during the same anodic cycle, whereas it mainly expanded at cathodic potentials in the first three cycles, but started to show mixed behavior in the last two. The actuation in Figure 7B exhibited a fully mixed behavior during the oxidation but barely expanded during the reduction. The third CP-yarn, Figure 7C, showed a mixed movement at anodic potentials and a pure cation-driven mechanism at cathodic potentials, similar to Figure 7A. These results suggest that the expansion and contraction of the CP-yarn are not purely cation- or anion-driven in this case either. Together with the solvent, both anions and cations are entering and leaving the matrix to balance the redox charge, and the actuation is the result of the absolute number of ions that go in or out. By using a well-known small and mobile anion in aqueous mixtures, single ion motion was pursued. Nevertheless, we obtained a mixed actuation mechanism. Therefore, other phenomena cause the alteration of the mechanism.^69,70^

Most of the literature studied the actuation of polypyrrole thin films that were polymerized on highly conductive metallic substrates. On the contrary, in our CP-based yarn actuators, the polypyrrole layer is deposited by electrosynthesis on a PEDOT:PSS layer. To date, the inner PEDOT:PSS layer was thought to be mainly responsible for electrical conductivity, only enabling the subsequent electropolymerization of a thick PPy actuating layer that covered the PEDOT layer with sufficient PPy. The mixed mechanism in the well-known anion-driven mechanism hinted toward the inner PEDOT layer also influencing the actuation mechanism. Due to the large size of the PSS molecule, the inner coating polymer PEDOT:PSS is known for exchanging cations.^71^ Therefore, the role of the underlaying PEDOT:PSS layer is investigated next: First, by enlarging the thickness of the PPy, and second, by replacing the PSS dopant with tosylate (Tos), known for exchanging anions instead of cations.^72^

### Effect of the Thickness of the PPy

In an attempt to block the possible cation exchange from the underlaying PEDOT:PSS layer, the thickness of the PPy layer was increased by prolonging the galvanostatic polymerization up to 20 000 and 30 000 s. A longer electropolymerization process generates a thicker layer of PPy. Figure S2 shows the chronopotentiograms of the PEDOT:PSS/PPy(ClO_4_)-yarns polymerized for 10 000, 20 000, and 30 000 s. They all presented the same potential behavior, matching the preceding polymerizations: a rapid first peak, followed by a valley and the final stabilization of the potential around 0.7 V. Figure S3 shows the cyclic voltammetry of different thicknesses of PPy(ClO_4_) compared to the PEDOT:PSS redox activity. CP-yarns subjected to longer polymerizations kept a constant potential and presented sufficient electroactivity after polymerization; CP-yarns with thicker layers of PPy showed higher reduction currents, which is attributed to greater amount of polypyrrole.^73^ However, PPy-based yarns subjected to longer polymerizations were easily cracked while being manipulated between the synthesis and actuation processes, preventing us from performing even longer electropolymerizations. It is worth mentioning that we did not consider decreasing the thickness of the PEDOT:PSS-coating, as this would have decreased the conductivity and subsequently impaired the electropolymerization conditions of PPy.

**Figure 8:**
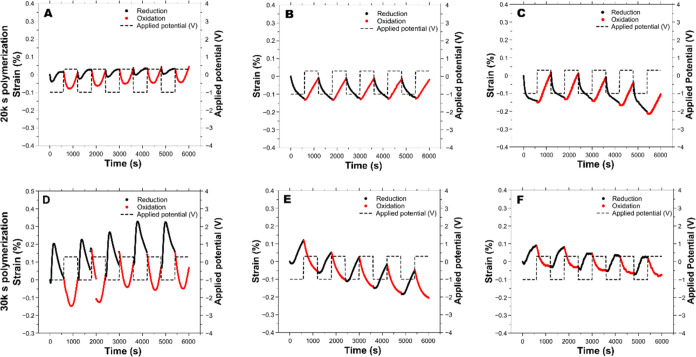
(A–C) Actuation response of PEDOT:PSS/PPy(ClO_4_) yarns synthesized for 20 000 s in 0.1 M LiClO_4_ aqueous solutions and actuated in 0.1 M LiClO_4_ aqueous. [−1, 0.3] V square wave potentials were applied at 0.83 mHz for 15 cycles. (D–F) Electrochemomechanical response of PEDOT:PSS/PPy(ClO_4_) yarns synthesized for 30 000 s in 0.1 M LiClO_4_ aqueous solutions and actuated in 0.1 M LiClO_4_ aqueous solutions by applying square wave potentials between [−1, 0.3] V at 0.83 mHz for 15 cycles.

Figure 8 shows the last five actuation cycles of the PPy(ClO_4_)-yarns electropolymerized for 20 000 and 30 000 s (see Figures S8 and S9 for the full actuation). Figure 8A–C shows the actuation of three different CP-yarn samples with 20 000 s long PPy electropolymerization. Figure 8D–F shows the actuation of three different CP-yarns with 30 000 s long PPy electropolymerization. Once again, different combinations of actuation mechanisms were obtained. The pure anion-driven actuation could finally be noticed for two of the three CP-yarns electropolymerized for 20 000 s (Figure 8B,C), contracting during reduction and expanding during oxidation. The latter might suggest that enlarging the thickness of the PPy layer is sufficient to block the cation motion from the underlaying PEDOT. However, yarns with thicker PPy layers (Figure 8E,F) showed a pure cation-driven actuation again; the thicker layer did not block the contribution of the PEDOT:PSS. Additionally, CP-yarns with thicker layers of PPy also presented mixed actuations (Figure 8A,D). Even though all of the samples were subjected to the same number of coatings and exhibited appropriate resistivities, slight differences were found in the linear density of the PEDOT:PSS-coating. CP-yarns presenting a pure anion-driven mechanism had the lowest PEDOT:PSS density, whereas the pure cation-driven samples had a thicker layer of PEDOT:PSS (150 vs 250 μg/cm), which can explain the difference between the results. The fibrous morphology of the yarns and the manual dip-coating method limit the control of homogeneous thickness along the CP-yarn.

Table 4 summarizes the average strain for CP-yarns polymerized for 10 000, 20 000, and 30 000 s in 0.1 M pyrrole and 0.1 M LiClO_4_ aqueous solution and actuated in the same but monomer-free electrolyte. In the case of mixed actuations, the maximum and minimum point were not reached at the end of the cycle but rather in the middle. Mixed mechanisms, like those shown in Figure 8A,D and previously in Figure 7, are undesirable not only because of their unpredictable behavior but also because of their low strain values.

**Table 4:** Comparison of the Effect of PPy Thickness on the Strain and Mechanism of CP-Yarns Electropolymerized for 10 000, 20 000, and 30 000 s^a^

combinations								
				actuation				
coating	polymerization of PPy					average strain		
polymer	time (s)	dopant	solvent	dopant	solv	reduction (%)	oxidation (%)	mechanism
PEDOT:PSS	10 000	ClO_4_^–^	AQ	LiClO_4_	AQ	0.16 ± 0.08	0.2 ± 0.05	mixed, cation-driven
PEDOT:PSS	20 000	ClO_4_^–^	AQ	LiClO_4_	AQ	0.17 ± 0.14	0.18 ± 0.13	mixed, anion-driven
PEDOT:PSS	30 000	ClO_4_^–^	AQ	LiClO_4_	AQ	0.087 ± 0.04	0.12 ± 0.04	mixed, cation-driven

a Solv represents solvent. AN and AQ stand for acetonitrile and aqueous solutions. C, M, and A represent cation-driven, mixed, and anion-dominant actuations. Errors in the average strain are calculated as the standard deviation of three actuations of three different samples.

Melling et al. demonstrated that thicker layers of PPy deposited on golden needles need more time to reach the maximum expansion; therefore, the strain could be lower if the length of the cycle is too short to reach the maximum expansion for thicker CP-yarns.^74^ The plateau in the actuation exhibited in Figure 7A suggests that CP-yarns polymerized for 10 000 s reached the maximum expansion within 300 s, whereas the sharp strain curves without a plateau suggest that the CP-yarns polymerized for 20 000 and 30 000 s did not reach the plateau values (Figure 8B–D). As a result, CP-yarns with thicker layers of PPy (20 000 and 30 000 s of polymerization) lead to slower mechanisms and lower the actuation strains. In any case, the cathodic expansion observed in well-known anion systems seems to be related to the redox activity of the underlaying PEDOT:PSS, which appears to be challenging to block by just enlarging the PPy film.

### Effect of the Dopant of PEDOT

Another way of removing the cation contribution of the underlying PEDOT:PSS layer is by replacing the bulky PSS dopant of the polymer with a mobile anion, such as tosylate (Tos). PEDOT(Tos) is a well-known anion exchange system.^75^ We coated the yarns with PEDOT(Tos) by vapor phase polymerization, and their resistivity was measured prior to the electropolymerization of PPy. CP-yarns coated with PEDOT(Tos) had, on average, 290 ± 58 Ω/cm resistivity. These CP-yarns were less conductive than PEDOT:PSS-coated CP-yarns, which resulted in a 120 ± 26 Ω/cm average resistivity. Thereafter, PPy was electropolymerized on PEDOT(Tos)-coated yarns. PEDOT(Tos)-PPy-coated yarns reached a similar but late plateau potential value during electropolymerization, when compared to PEDOT:PSS–PPy-coated yarns (see Figure S10). Due to conductivity differences, the range of the cyclic voltammetry performed after the electropolymerization was expanded to −1.2 and 0.5 V and swiped at the same 10 mV/s speed (Figure S10). As a result, the current peaks were shifted to new limits of the wider potential window, which means that the maximum redox speed will not be reached in the following chronoamperometry, in which the reduction and oxidation limits are kept between −1 and 0.3 V, to be in concordance with the previous experiments.

**Figure 9:**
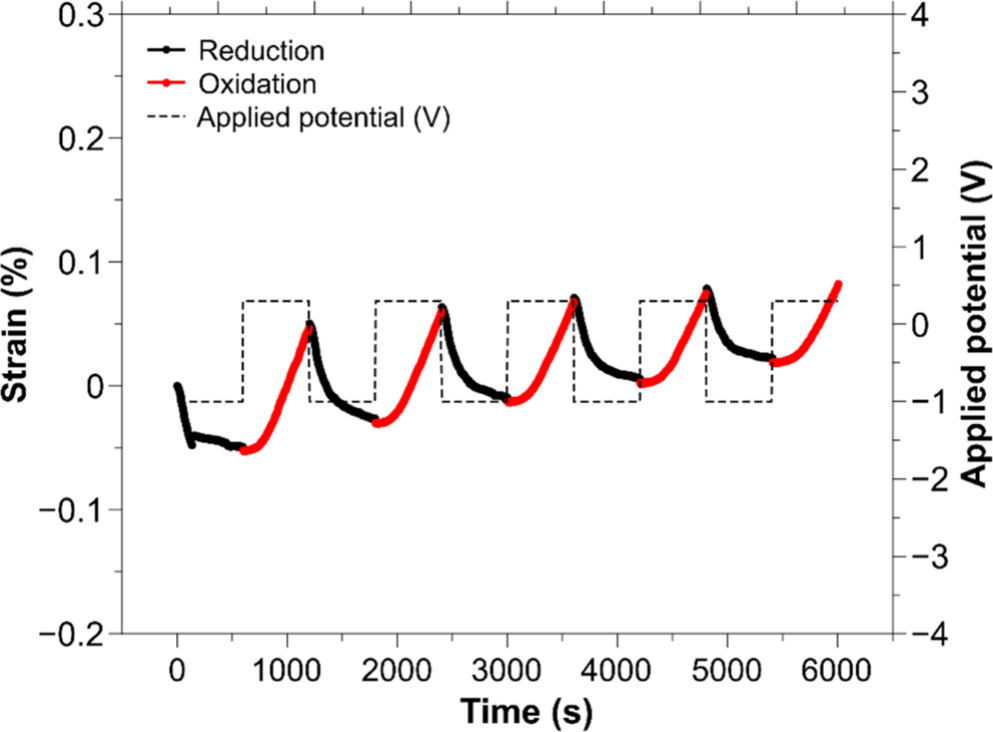
Actuation of PEDOT(Tos)/PPy(ClO_4_) yarns doped for 10 000 s in 0.1 M LiClO_4_ aqueous solutions and actuated in 0.1 M LiClO_4_ aqueous solutions. [−1, 0.3] V square wave potentials were applied at 0.83 mHz for 15 cycles.

PEDOT(Tos)/PPy(ClO_4_)-yarns were fabricated and subjected to chronoamperometry between −1 and 0.3 V for 15 cycles, and the isotonic strain was measured. Figure 9 shows the last 5 cycles out of 15 and the typical anion-dominant actuation mechanism can now be clearly seen, which was obtained in all of the repetitions of the yarn actuators with the underlaying PEDOT(Tos)-coating.

These results indicate that the underlaying PEDOT:PSS was responsible for exchanging the cations and, herewith, switching the mechanism. The underlaying PEDOT:PSS layer influenced not only the electrochemical responses but also its actuation mechanism.^59^ However, the strain of anion-driven actuation was decreased. The CP-yarns contracted and expended on average by 0.1% (Table 5), 10-fold less than the maximum strain we achieved in this work using PEDOT:PSS/PPy. As predicted in the cyclic voltammetry, sharp actuation peaks during the oxidation suggest that the oxidation speed is slow (or longer potential steps are needed) for the chosen potential limits. Applying a wider voltage range might increase the redox charge and, thereby, the strain values.

**Table 5:** Comparison of the Effect of the Inner PEDOT Layer on the Strain and Mechanism of CP-Yarns^a^

combinations								
				actuation				
coating	polymerization of PPy					average strain		
polymer	time (s)	dopant	solv	dopant	solv	reduction (%)	oxidation (%)	mechanism
PEDOT:PSS	10 000	ClO_4_^–^	AQ	LiClO_4_	AQ	0.16 ± 0.08	0.23 ± 0.03	mixed, cation-driven
PEDOT(Tos)	10 000	ClO_4_^–^	AQ	LiClO_4_	AQ	0.06 ± 0.05	0.08 ± 0.04	anion-driven

a Solv represents solvent, Red and Ox mean reduction and oxidation, and mech corresponds to the actuation mechanism. AN and AQ stand for acetonitrile and aqueous solutions. C, M, and A represent cation-driven, mixed, and anion-dominant actuations. Errors in the average strain are calculated as the standard deviation of three actuations of three different samples.

Contrary to our hypothesis, none of the yarns actuated in EMImOTf acetonitrile or aqueous solutions showed a pure anion-driven actuation mechanism, not even when PEDOT(Tos)/PPy(ClO_4_) yarns were actuated in EMImOTf aqueous solution (Figure S13). In this work, the dual-actuation system was possible only if the anion-driven CP-yarn was PEDOT(Tos)/PPy(ClO_4_) actuated in LiClO_4_ aqueous solution. Even though the EMImOTf ionic liquid has shown great characteristics to prepare ionogels, such gels can also be prepared with LiClO_4_ salts.^76^ The latter is important if these systems are to be used as in-air operating actuators. Next, in order to pair the PEDOT(Tos)/PPy(ClO_4_) yarns with the cation-driven PEDOT:PSS/PPy(DBS) actuated in LiClO_4_, the cation-driven CP-yarns were tested in the same electrolyte.

### Dual Actuation of CP-Yarns

In the second part of this work, we demonstrate the dual actuation of two CP-yarns driven by two yarns that were doped with small and large anions, respectively, and led to concurrent actuation mechanisms upon opposite redox reactions. Based on the results displayed in the first part, the anion-driven CP-yarn was prepared by vapor-phase polymerizing PEDOT(Tos) on the yarn and electropolymerizing PPy(ClO_4_) on top of it. For the cation-driven CP-yarn, the yarn was dip-coated in PEDOT:PSS and PPy(DBS) was electropolymerized on top. Since pure anion-driven actuations were only obtained by actuating the PEDOT(Tos)/PPy(ClO_4_) CP-yarn in LiClO_4_ aqueous solution, the dual actuation was performed in the same electrolyte. Prior to that, we checked the actuation of the PEDOT:PSS/PPy(DBS) CP-yarn in that solution.

**Figure 10:**
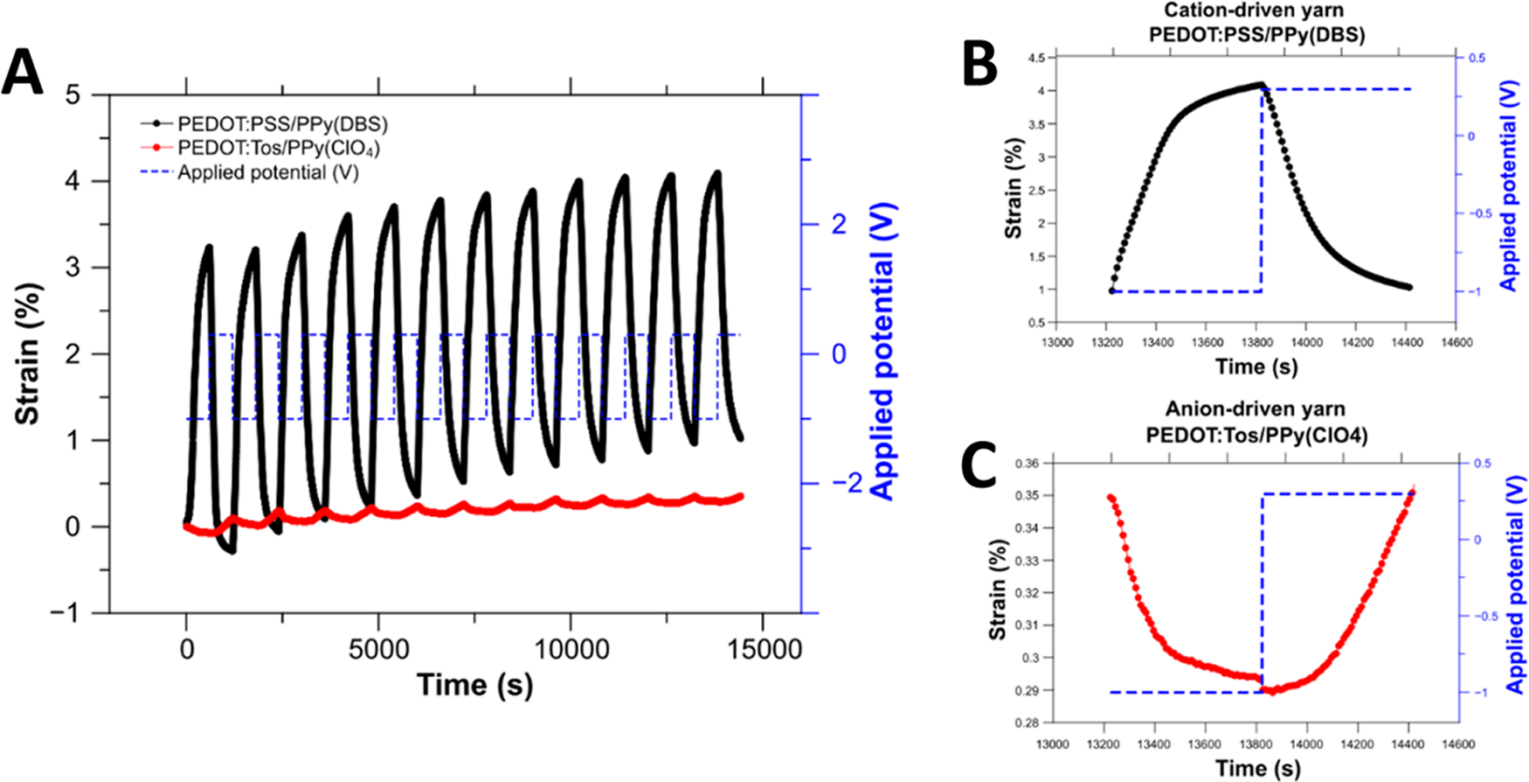
(A) Actuation of the PEDOT:PSS/PPy(DBS)-coated yarn in LiClO_4_ aqueous solution (black). The actuation of PEDOT(Tos)/PPy(ClO_4_)-coated yarn in a LiClO_4_ aqueous solution (red). Applied potential of [−1, 0.3] V for 600 s at each potential (blue dashed line). (B) Single cycle of the cation-driven actuation of PEDOT:PSS/PPy(DBS)-coated yarn (black) and applied potential (blue dashed line). (C) Single cycle of the anion-driven actuation of PEDOT(Tos)/PPy(ClO_4_)-coated yarn (red) and the applied potential (blue dashed line).

Figure 10A shows the actuation of the single cation-driven PEDOT:PSS/PPy(DBS) CP-yarn in a LiClO_4_ aqueous solution (black) compared to the single anion-driven PEDOT(Tos)/PPy(ClO_4_) CP-yarn in the same electrolyte (red). The cation-driven CP-yarn showed the highest strain reported so far for CP-yarn actuators, a maximum of 3.2% for one of the samples. The average of the three samples was 1.9 ± 1.2%. The average of the cation-driven yarn is almost 20-fold greater than the average strain obtained with the anion-driven yarn, 0.1%. Figure 10B shows a single cycle of PEDOT:PSS/PPy(DBS)-yarn, where small Li^+^ cations enter the polymer upon reduction (expansion) and leave upon oxidation (contraction). Figure 10C shows a single cycle of the PEDOT(Tos)/PPy(ClO_4_)-yarn, which shows the reverse behavior, i.e., ClO_4_^–^ exits the polymer upon reduction (contraction) and enters upon oxidation. (expansion). Even though the difference between anion-driven and cation-driven CP-yarns remains quite big, the yarns actuate concurrently in the same direction.

**Figure 11:**
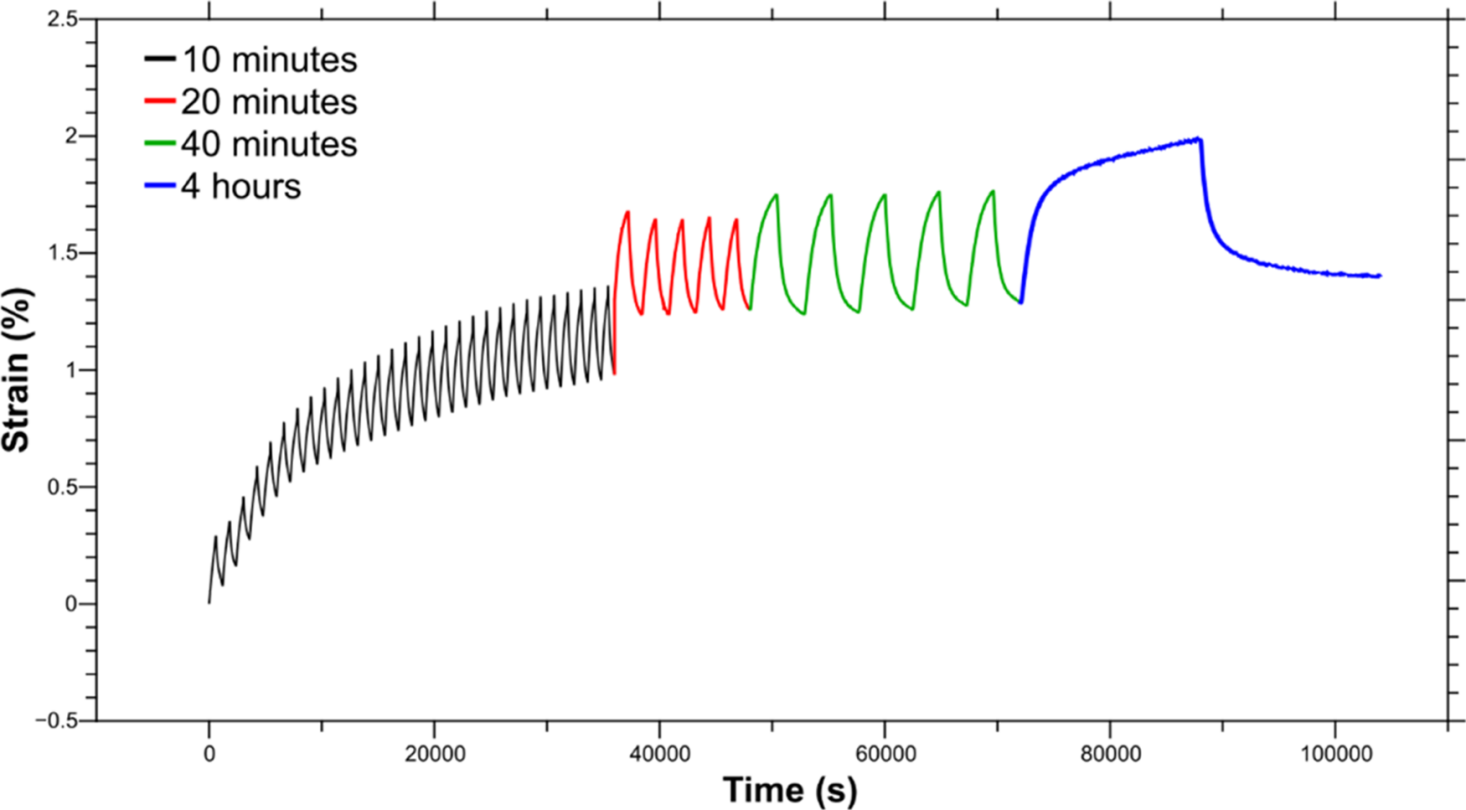
Dual actuation of PEDOT:Tos/PPy(ClO_4_)-coated yarn and PEDOT PEDOT:PSS/PPy(DBS)-coated yarn in LiClO_4_ aqueous solution. Applied potential of [−1,1] V for 600 s (10 min, black), 1200 s (20 min, blue), 2400 s (40 min, green), and 16000 s (4 h, blue) at each potential.

Finally, the anion-driven and cation-driven CP-yarns were simultaneously actuated in the two-electrode configuration. The cation-driven yarn is connected as the working electrode and the cation-driven yarn is connected as the counter electrode, short-circuited with the reference electrode (Figure 2, step 3B). Figure 11 shows the dual actuation of the system for 10 min, 20 min, 40 min, and 4 h long cycles using a symmetric [−1, 1] V potential range. The actuation is stable for over 100 000 s and reaches saturation during the oxidation after a 4 h long cycle. The dual actuation was repeated three times with three different samples for each type of yarn and reached an average of 0.33 ± 0.06% in the first 10 min, 0.39 ± 0.00% after 20 min, 0.46 ± 0.02% upon 40 min, and a maximum of 0.56 ± 0.01% after 4 h at each potential limit (Table 6). 80% of the maximum actuation strain is reached within the first 40 min, then the speed of the actuation decreases, and the cycle needs to be prolonged over 3 h to gain additional 0.1% of actuation. The strain of the dual-actuation system falls somewhere in between the strains obtained for the individual single yarns for the same cycle durations (10 min). The dual actuations’ 0.33% strain is 6-fold lower compared to the cation-driven single-yarn actuation, but 3 times greater than the anion-driven single-yarn actuation. The anion-driven yarn is mechanically impeding the actuation of the cation-driven yarn, yet it is not counter acting like other systems proposed in the past.^48^

**Table 6:** Strain and Mechanism of the Dual Actuation in a LiClO_4_ Aqueous Solution of Two Yarns: CP-Yarns Were Coated with PEDOT:PSS and PPy Doped with DBS^–^ Anions and PEDOT:Tos and PPy Doped with ClO_4_^–^ Anions^a^

				actuation					
coating	polymerization						average strain		
polymer	time (s)	dopant	solv	electrolyte	solv	time at each limit	reduction (%)	oxidation (%)	mechanism
PEDOT:PSS	10 000	DBS^–^	AQ	LiClO_4_	AQ	10 min	0.33 ± 0.06	0.32 ± 0.06	dual mechanism
						20 min	0.39 ± 0.00	0.38 ± 0.00	
PEDOT(Tos)	10 000	ClO_4_^–^	AQ			40 min	0.46 ± 0.02	0.46 ± 0.02	
						4 h	0.56 ± 0.01	0.47 ± 0.22	

a Solv stands for the solvent. AQ represents aqueous solutions. Errors in the average strain are calculated as the standard deviation of three actuations of three different samples.

We believe there are more reasons, in addition to the mechanical aspect, that hinder the dual actuation. In an electrochemical cell, the current is proportional to the rate of reactions in the system according to Faraday’s law of electrolysis, *F* = *nF*ν, where *n* is the stoichiometric number of electrons in the balanced redox equation (in mol), *F* is the Faraday constant (96 485 C/mol), and ν is the rate of the electrochemical reaction (in moles per second). In the dual actuation, the stoichiometric numbers of electrons for the redox reactions at the anode and cathode are the same. Therefore, the reaction in the electrode with the slowest rate of reaction will be the limiting step. In this case, based on the single-yarn results, we believe that the anion-driven system’s rate is the slowest one, lowering the current and hindering the actuation by slowing it down. Also, we believe that the total charge exchanged in the dual actuation is limited. Table 7 shows the consumed charge of PEDOT:PSS/PPy(DBS)- and PEDOT:Tos/PPy(ClO_4_)-yarns when actuated as a single yarn in LiClO_4_ aqueous solutions in a three-electrode-cell configuration (columns 1 and 2) and the consumed charge of the dual-actuation system, i.e., PEDOT:PSS/PPy(DBS)-yarn and PEDOT:Tos/PPy(ClO_4_)-yarn actuated in a two-electrode-cell configuration (column 3). Note also that the potentials applied in the dual actuation were symmetric [−1, 1] V versus the opposite yarn, in contrast to the potential limits applied in the single-yarn experiments [−1, 0.3] V, versus the Ag/AgCl reference electrode. On average, for single PEDOT:PSS/PPy(DBS)-yarns, the total reduction and oxidation charges during the actuation were −190 ± 70 and 170 ± 70 mC, respectively. For single PEDOT:Tos/PPy(ClO_4_)-yarns, the total reduction and oxidation charges were −38 ± 10 and 30 ± 10 mC. From the charge evolution shown in Figure S12, where the strain has not yet reached a plateau, we conclude that the PEDOT:Tos/PPy(ClO_4_)-yarn is not yet fully oxidized or reduced since the charge evolution could still increase if the experiment would had been longer, indicating that the charging capacity of this yarn is higher. In contrast, PEDOT:PSS/PPy(DBS)-yarns reached a plateau in the charge evolution (Figure S14), displaying that the CP reached its maximum capacity of charging. The latter explains why in the dual actuation we achieved a higher charging capacity than in the single anion-driven yarn. However, as predicted, the dual actuation is limited by the anion-driven yarn, since its charge is still half the charge capacity of the cation-driven yarn.

**Table 7:** Consumed Charge by Single-Yarn PEDOT:PSS/PPy(DBS) and PEDOT:Tos/PPy(ClO_4_) during the Oxidation and Reduction When Applying [−1, 0.3] V for 10 min at Each Potential and in the Dual-Actuation System When Applying [−1, 1] V for 10 min at Each Potential^a^

	PEDOT:PSS/PPy(DBS)	PEDOT(Tos)/PPy(ClO_4_)	dual actuation
*Q*_oxidation_ (mC)	170 ± 70	30 ± 10	76 ± 14
*Q*_reduction_ (mC)	–190 ± 70	–38 ± 10	–80 ± 20

a All yarns are actuated in 0.1 M LiClO_4_ aqueous solution and had an active length of 20 mm.

## Conclusions

The objective of this work was to demonstrate the possibility of performing dual actuation of two CP-yarns, one doped with small anions and the other one doped with large anions, that would expand and contract simultaneously upon opposite redox reactions, both contributing to an efficient movement. This configuration enables the possibility of removing the external counter electrode of the system, leading to more compact systems, as needed for in-air-operated yarn actuators.

In the first part of this paper, we focused on investigating CP-yarns that could actuate according to the anion-dominated mechanism. For this, we studied the effect of the actuation electrolyte, the dopants of the PPy, the solvent, the thickness of the PPy, and the dopant of the PEDOT layer on the actuation strain and mechanism of the CP-yarns. We have shown that, from all of the systems that we tested, the only possible way of achieving an anion-driven CP-yarn was by having both PEDOT and PPy layers doped with small anions, Tos and ClO_4_^–^, respectively. The latter CP-yarn showed an average strain of 0.1%, which was 20-fold lower than the cation-driven PEDOT:PSS/PPy(ClO_4_)-yarn, which showed an average strain of 1.9 ± 1.2%. These results also showed that, contrary to our hypothesis, the PEDOT layer not only functions as the conductive coating for the nonconductive yarns but also plays an active role in the actuation mechanism of the CP-yarn.

In the second part of this article, we have combined anion-driven and cation-driven CP-yarns to create dual actuation. Based on the results of the first part, a dual yarn actuator was constructed consisting of PEDOT:PSS/PPy(DBS) CP-yarn for the cation-driven half, PEDOT(Tos)/PPy(ClO_4_) for the anion-driven half, and LiClO_4_ aqueous solution for the actuation electrolyte. The dual actuation showed a strain of 0.33 ± 0.06% upon 10 min at each potential limit, and up to 0.56 ± 0.01% when each potential limit is applied for 4 h. The dual CP-yarns also exhibit remarkable stability during a total of 41 cycles and almost 28 h of actuation.

To conclude, this study will help the development of linear textile actuators by better understanding the effect of the electrolyte, solvent, and polymers on the actuation results. By tailoring the different parameters and understanding their effect in the system, we will be able to design actuators that move upon an anion-driven mechanism or, alternatively, cation-driven. This will enable the construction of dual mechanism linear in-air yarn actuators comprising the two electrodes needed to drive the electrochemical reaction by just using yarns. Such in-air yarn actuators will consecutively be woven or knitted into electromechanically active fabrics enabling garments that can provide mechanical stimulation for, e.g., haptic garments for physical training, gaming, tactical communication equipment in military, navigation for visually impaired people, and soft exoskeletons for supporting garments augmenting people working within heavy load carriers, among others.

## Supporting Information Available

Electropolymerization, cyclic voltammetry, and full actuation supporting graphs mentioned in the Results and Discussion section, as well as a table with the strain and charge values from all three repetitions used to calculate the average strain and charge values presented in the main text (PDF)
