# Supplementary material for: Synchronous Cation-Driven and Anion-Driven Polypyrrole-Based Yarns toward In-Air Linear Actuators
Source: Chem Mater. 2024 Sep 30;36(19):9391–405. doi: 10.1021/acs.chemmater.4c00873 (PMC11467901; doi:10.1021/acs.chemmater.4c00873)
Supplement: Supplementary file 1 — cm4c00873_si_001.pdf [file cm4c00873_si_001.pdf]

Supporting Information

Synchronous cation-driven and anion-driven polypyrrole-based yarns towards in-air linear actuators

Amaia B. Ortega-Santos, Jose G. Martínez, Edwin W. H. Jager

SI: Results and Discussion

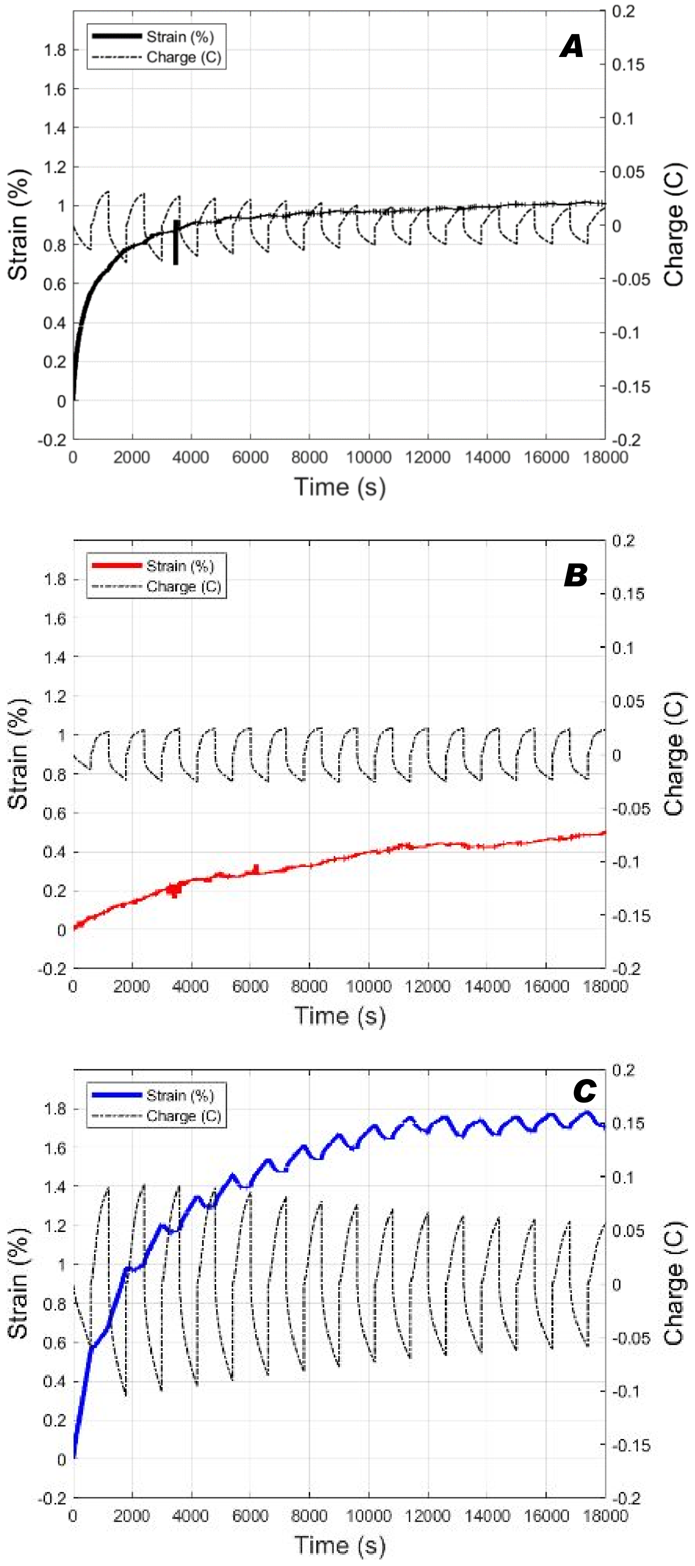

Figure S1: Full actuation of PEDOT:PSS/PPy(DBS)-yarns' actuation. [-1, 0.3] V square wave potential was applied at 0.83 mHz in 0.1 M NaOTf (A), TBAOTf(B), and EMImOTf(C) acetonitrile solution.

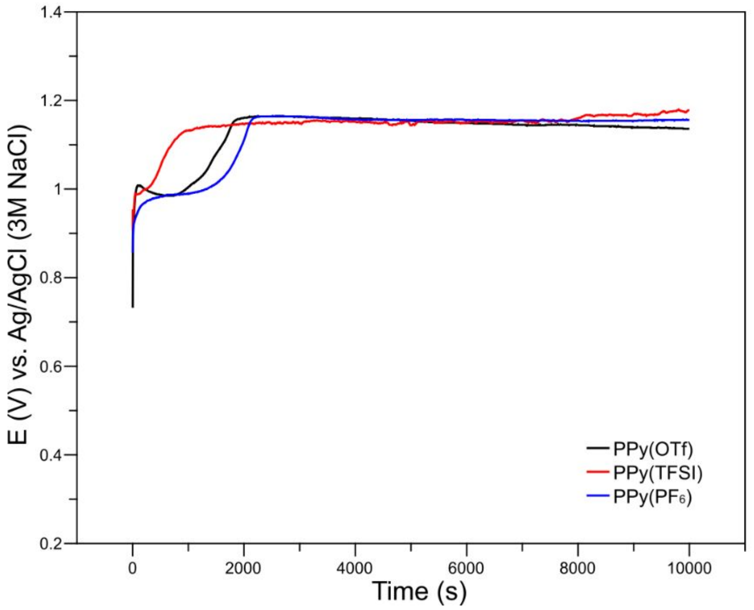

Figure S2: Chronopotentiograms of the electropolymerisation of PPy onto the PEDOT:PSS layer in 0.1 M TBAOTf, TBAPF$_6$, and LiTFSI acetonitrile solutions. The current was set to 0.5 mA for 10000 s.

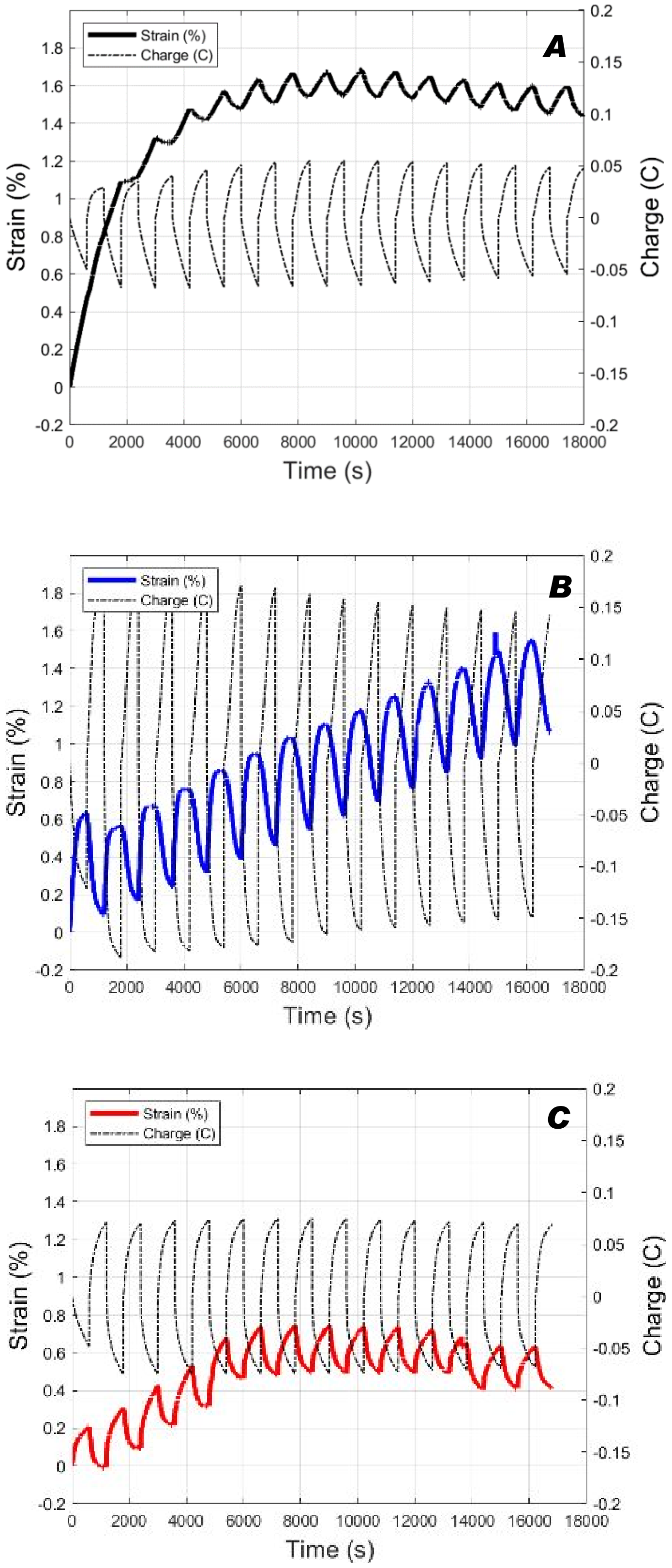

Figure S3: Full actuation of PPy(TFSI)-, PPy(OTf)-, and PPy(PF_6)-yarns actuation last five cycles. Square wave potential was applied between [-1, 0.3] V against Ag/AgCl (3 M NaCl) at 0.83 mHz in 0.1 M EMImOTf acetonitrile solution.

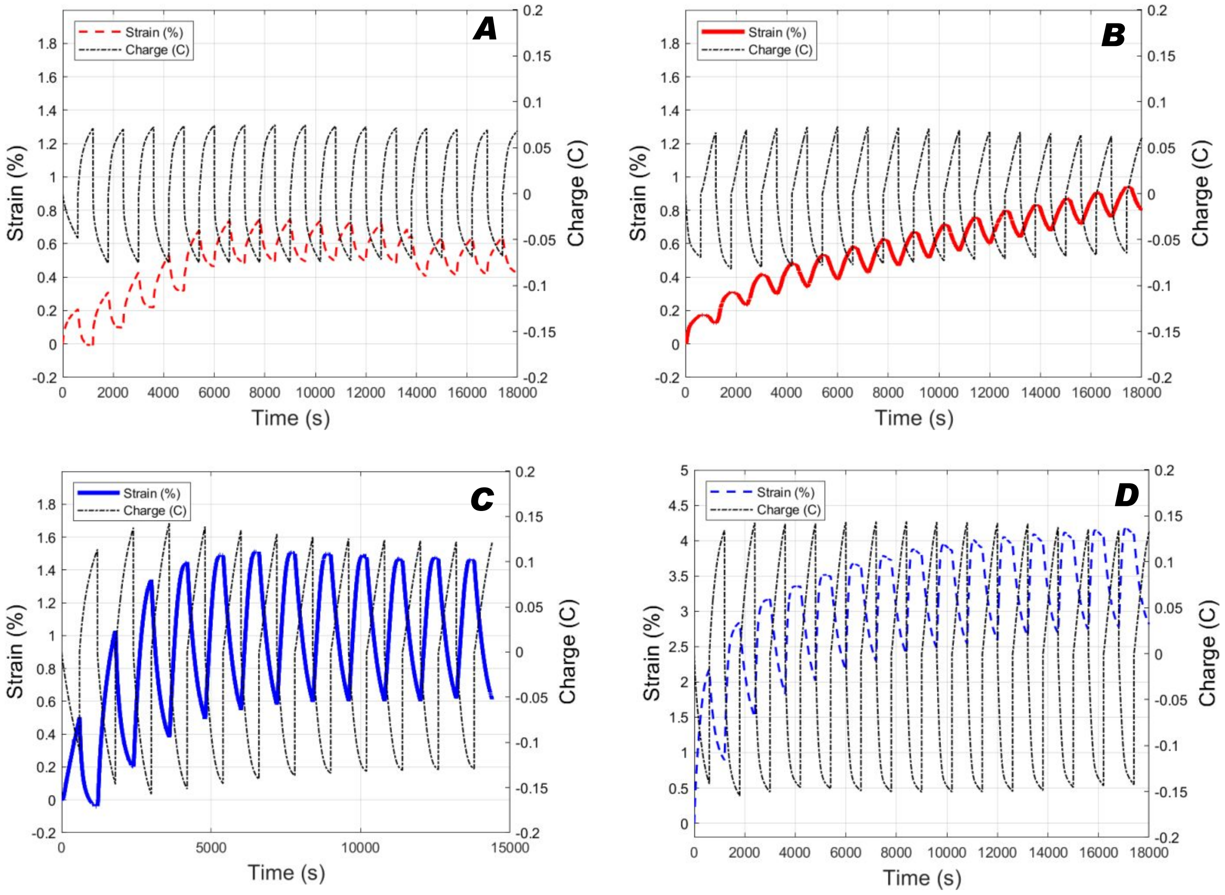

Figure S4: Full actuation of PEDOT:PSS/PPy(TFSI)-yarns. (A) Electropolymerization and doping in 0.1 M pyrrole, 0.1 M LiTFSI acetonitrile mixture and actuation in 0.1 M LiTFSI acetonitrile solution. (B) Electropolymerization and doping in 0.1 M pyrrole, 0.1 M LiTFSI aqueous mixture and actuation in 0.1 M LiTFSI acetonitrile solution. (C) Electropolymerization and doping in 0.1 M pyrrole, 0.1 M LiTFSI aqueous and actuation in 0.1 M LiTFSI aqueous solution. (D) Electropolymerization and doping in 0.1 M pyrrole, 0.1 M LiTFSI acetonitrile and actuation in 0.1 M LiTFSI aqueous solution. Square wave potential was applied between [-1, 0.3] V against Ag/AgCl (3 M NaCl) at 0.83 mHz in 0.1 M EMImOTf acetonitrile solution.

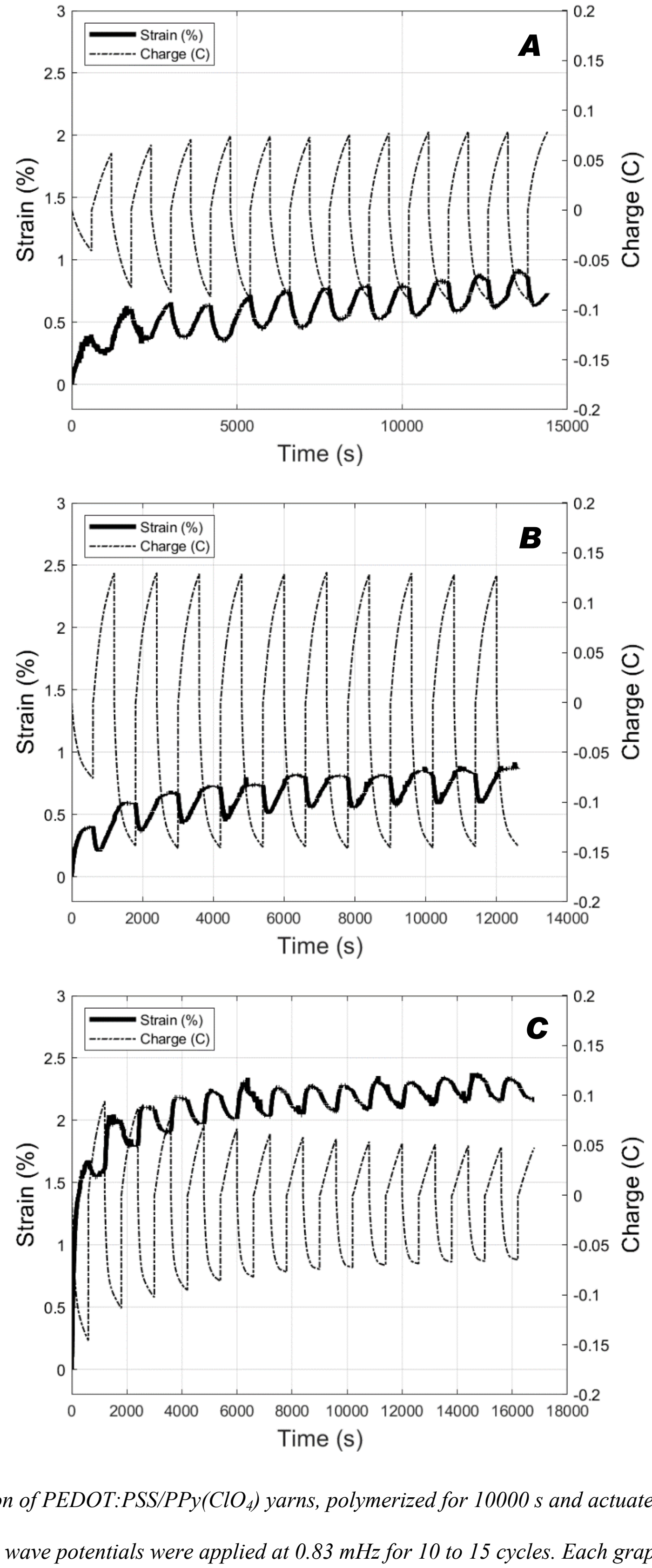

Figure S5: The full actuation of PEDOT:PSS/PPy(ClO_4) yarns, polymerized for 10000 s and actuated in 0.1 M $LiClO_4$ aqueous. [-1, 0.3] V square wave potentials were applied at 0.83 mHz for 10 to 15 cycles. Each graph represents one out of the three repetitions that were performed.

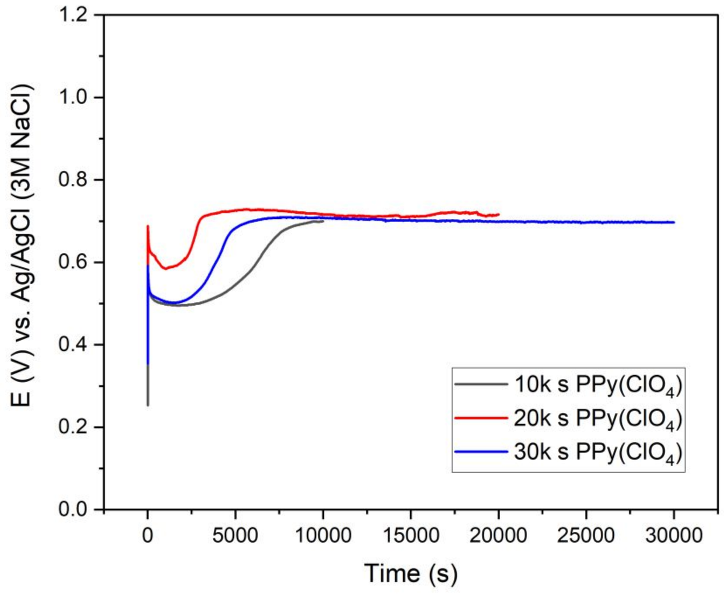

Figure S6: Chronopotentiograms of the electropolymerisation of PPy(ClO_4) onto the PEDOT:PSS layer for 10000, 20000, and 30000 s. The current was set to 0.5 mA.

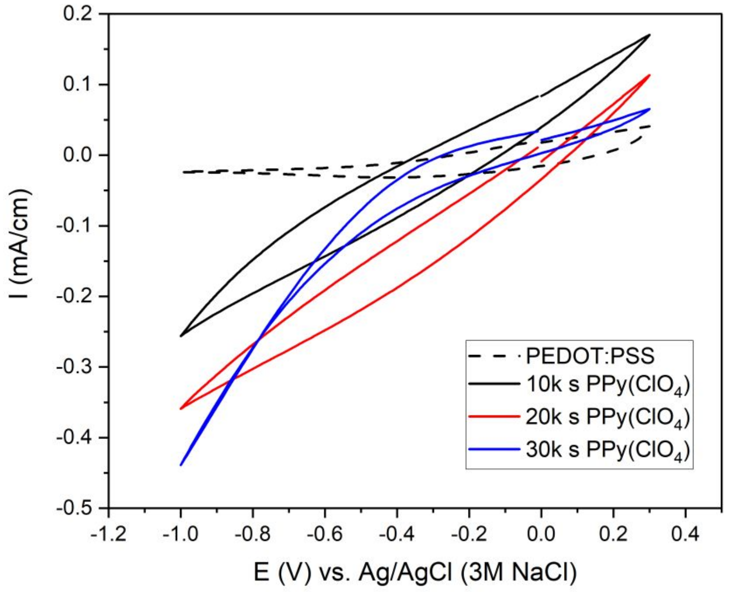

Figure S7: The redox activity of the yarns polymerised for 10000, 20000, and 30000 s in 0.1 M $LiClO_4$ aqueous solutions. The potential was swiped between [-1, 0.3] V at 10 mV/s, three times. The last of those 3 cycles is shown here.

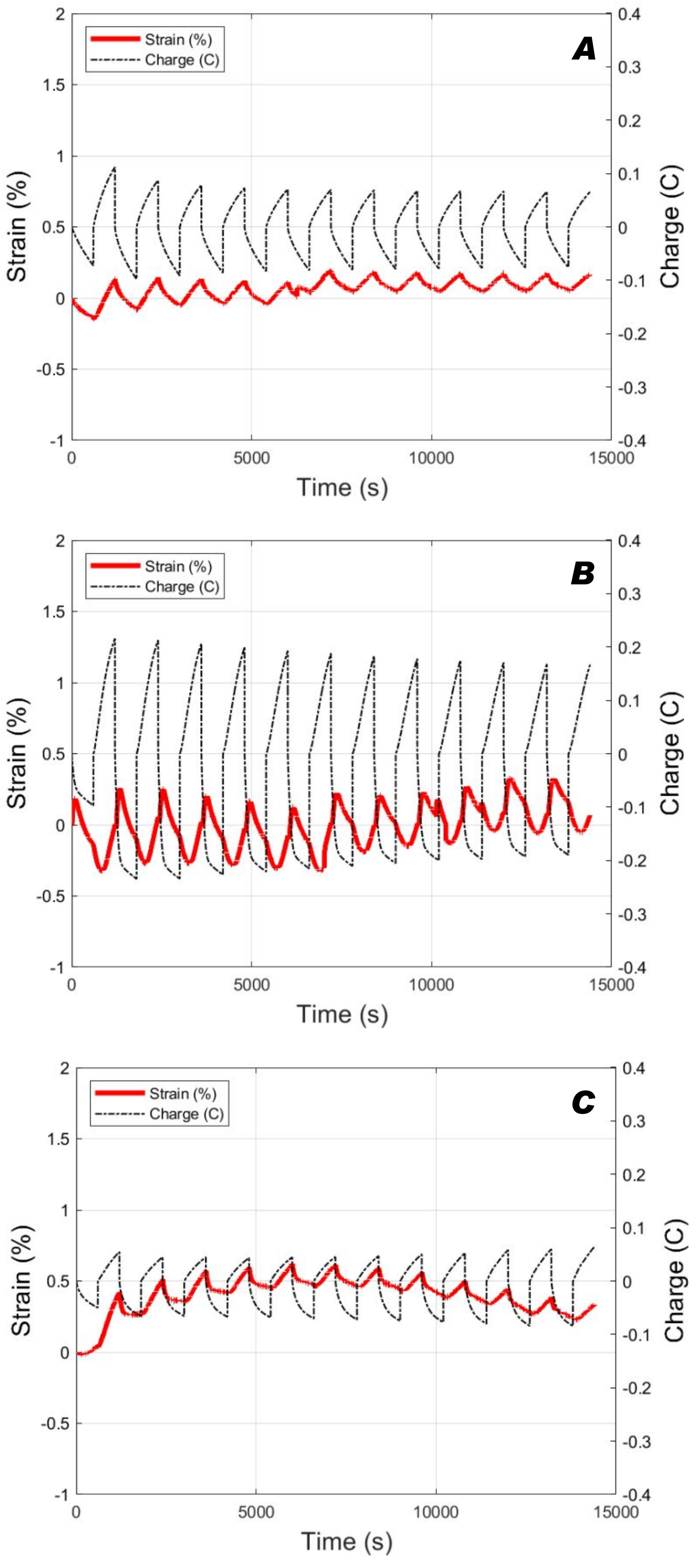

Figure S8: The full actuation of PEDOT:PSS/PPy(ClO_4) yarns, polymerized for 20000 s and actuated in 0.1 M $LiClO_4$ aqueous. [-1, 0.3] V square wave potentials were applied at 0.83 mHz for 10 to 15 cycles. Each graph represents one out of the three repetitions that were performed.

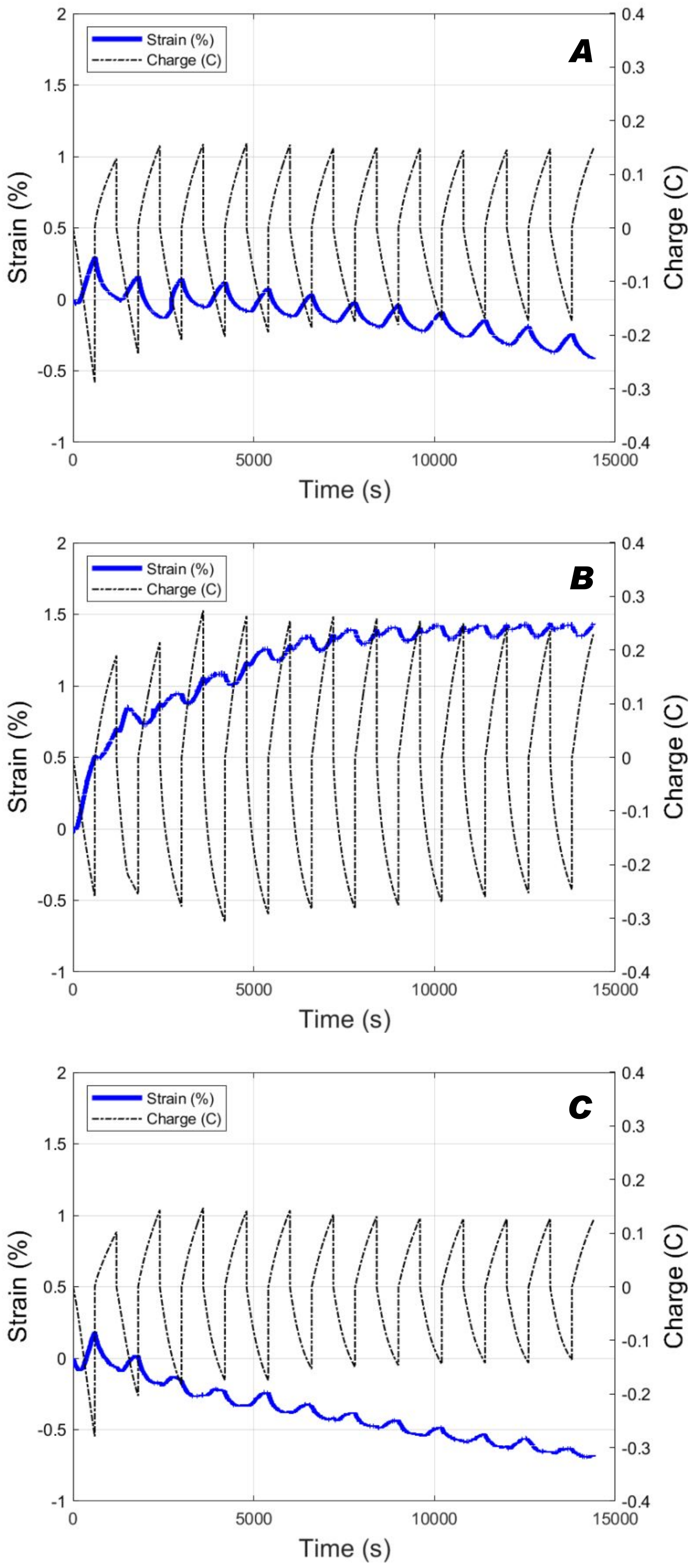

A
Strain (%)
Charge (C)
Time (s)
B
C

Figure S9: The full actuation of PEDOT:PSS/PPy(ClO_4) yarns, polymerized for 30000 s and actuated in 0.1 M $LiClO_4$ aqueous. [-1, 0.3] V square wave potentials were applied at 0.83 mHz for 10 to 15 cycles. Each graph represents one out of the three repetitions that were performed.

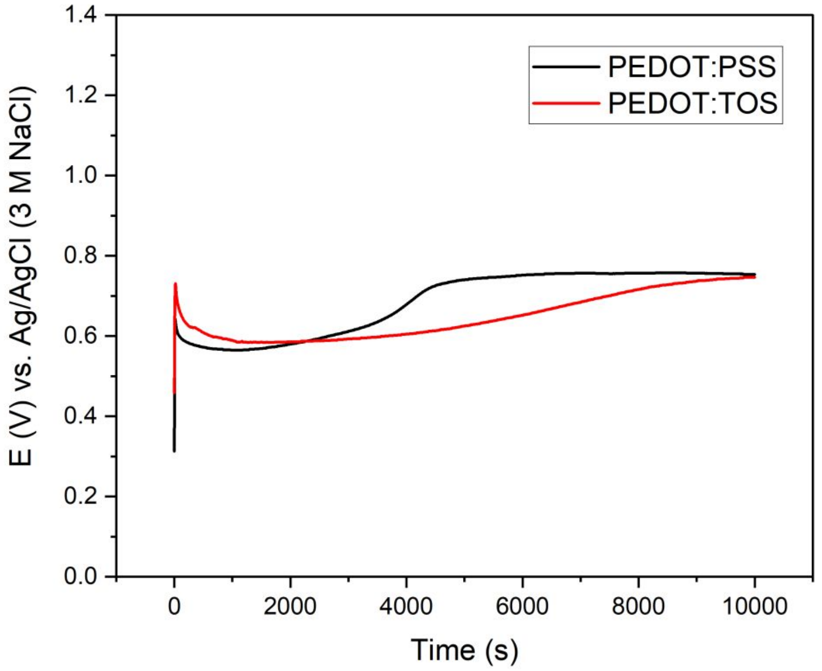

Figure S10: Chronopotentiograms of the electropolymerisation of PPy(ClO_4) onto the PEDOT:PSS and the PEDOT(Tos) layer. The current was set to 0.5 mA for 10000 s.

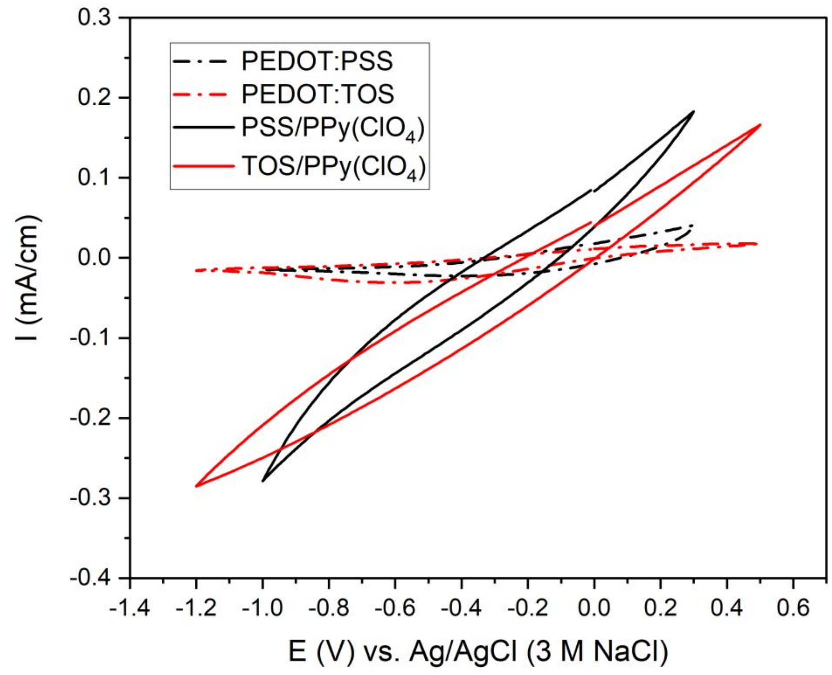

Figure S11: Cyclic voltammetries of the PPy(ClO_4) before and after the polymerization. The potential was swiped between [-1.2, 0.5 V] for the CP- yarns coated with PEDOT(Tos) and between [-1, 0.3] V for the CP-yarns coated with PEDOT:PSS. All the cyclic voltammetries were performed at 10 mV/s for 3 cycles. The last of those 3 cycles is shown here.

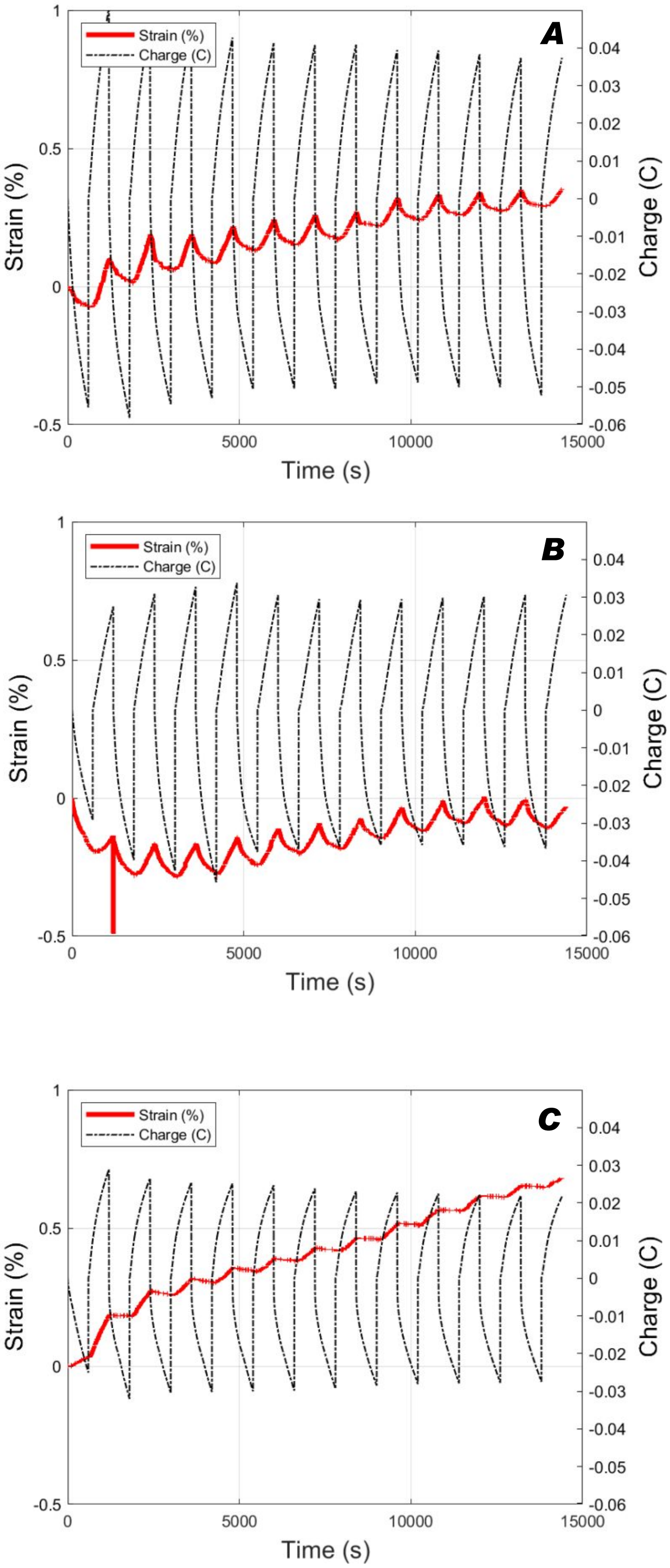
A
Strain (%)
Charge (C)
Time (s)
B
Strain (%)
Charge (C)
Time (s)
C
Strain (%)
Charge (C)
Time (s)

Figure S12: The full actuation and charge of PEDOT:TOS/PPy(ClO$_4$) yarns actuated in 0.1 M LiClO$_4$ aqueous solution. [-1, 0.3] V square wave potentials were applied at 0.83 mHz for 10 to 15 cycles. Each graph represents one out of the three repetitions that were performed.

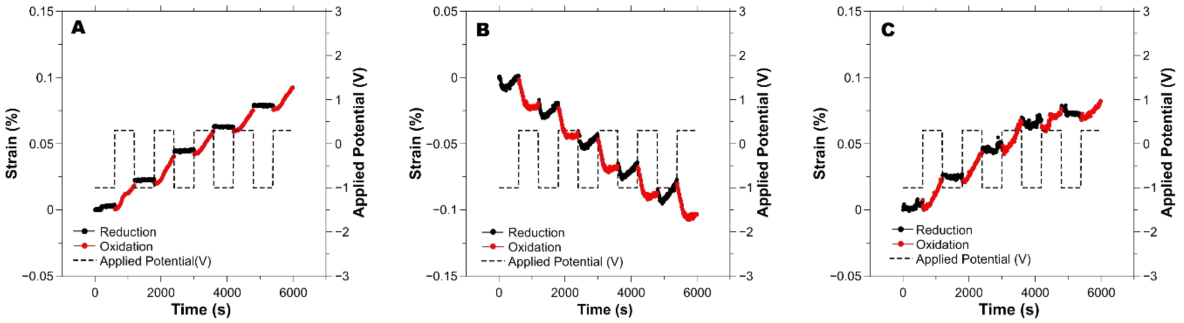

Figure S13: The last 5 cycles of the actuation of PEDOT:TOS/PPy(ClO$_4$) yarns actuated in 0.1 M EMImOTf aqueous solution. [-1, 0.3] V square wave potentials were applied at 0.83 mHz for 10 to 15 cycles. Each graph represents one out of the three repetitions that were performed.

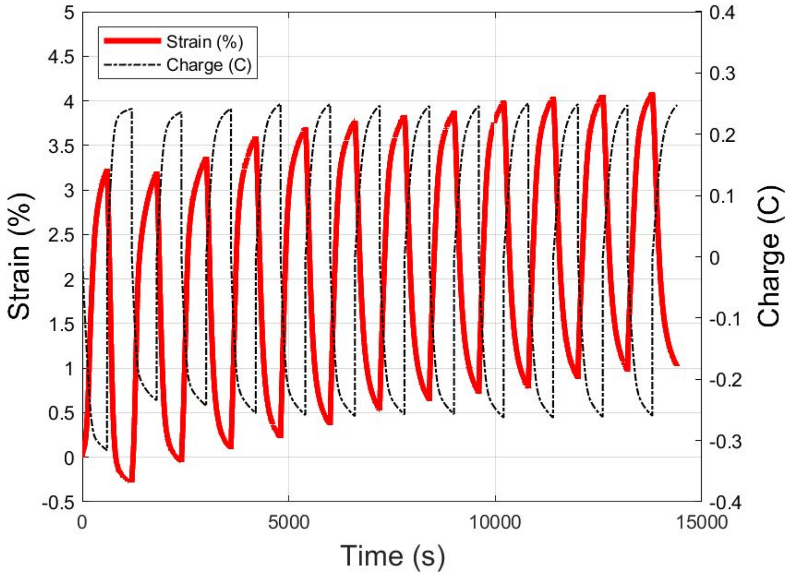

Figure S14: The actuation and charge of PEDOT:PSS/PPy(DBS) coated yarn in LiClO$_4$ aqueous solution (red). Applied potential [-1, 0.3] V for 600 s at each potential.

Experiment	Sample	PEDOT	PPy(A)	Poly solv	Poly t	Act salt	Act solv	Applied pot	t at each limit	Strain Red	Strain Ox	Charge Red	Charge Ox	Irreversible charge	Degradation Red	Degradation Ox
					s			V	s	%	%	C	C	%	%	%
Effect of the actuation Salt	a	PEDOT:PSS	PPy(DBS)	AQ	10k	EMImOTf	AN	[-1,0.3]	600	0.09%	0.05%	-0.070	0.067	5%	9%	8%
	b	PEDOT:PSS	PPy(DBS)	AQ	10k	EMImOTf	AN	[-1,0.3]	600	0.11%	0.00%	-0.061	0.061	1%	15%	11%
	c	PEDOT:PSS	PPy(DBS)	AQ	10k	EMImOTf	AN	[-1,0.3]	600	0.09%	0.08%	-0.063	0.060	4%	39%	37%
	a	PEDOT:PSS	PPy(DBS)	AQ	10k	NaOTf	AN	[-1,0.3]	600	0.01%	0.02%	-0.033	0.031	6%	14%	6%
	b	PEDOT:PSS	PPy(DBS)	AQ	10k	NaOTf	AN	[-1,0.3]	600	0.01%	0.01%	-0.024	0.024	1%	9%	3%
	c	PEDOT:PSS	PPy(DBS)	AQ	10k	NaOTf	AN	[-1,0.3]	600	0.01%	0.00%	-0.018	0.019	-3%	42%	37%
	a	PEDOT:PSS	PPy(DBS)	AQ	10k	TBAOTf	AN	[-1,0.3]	600	0.02%	0.01%	-0.030	0.026	15%	36%	31%
	b	PEDOT:PSS	PPy(DBS)	AQ	10k	TBAOTf	AN	[-1,0.3]	600	0.02%	0.01%	-0.024	0.024	1%	46%	44%
	c	PEDOT:PSS	PPy(DBS)	AQ	10k	TBAOTf	AN	[-1,0.3]	600	0.02%	0.02%	-0.018	0.016	8%	39%	37%
Effect of the PPy dopant	a	PEDOT:PSS	PPy(OTf)	AN	10k	EMImOTf	AN	[-1,0.3]	600	0.02%	0.01%	-0.061	0.055	10%	-8%	-41%
	b	PEDOT:PSS	PPy(OTf)	AN	10k	EMImOTf	AN	[-1,0.3]	600	0.13%	0.15%	-0.059	0.050	17%	20%	-3%
	c	PEDOT:PSS	PPy(OTf)	AN	10k	EMImOTf	AN	[-1,0.3]	600	0.01%	0.01%	-0.017	0.010	65%	73%	52%
	a	PEDOT:PSS	PPy(PF_6)	AN	10k	EMImOTf	AN	[-1,0.3]	600	0.24%	0.21%	-0.074	0.070	6%	39%	36%
	b	PEDOT:PSS	PPy(PF_6)	AN	10k	EMImOTf	AN	[-1,0.3]	600	0.22%	0.19%	-0.049	0.047	3%	-49%	-68%
	c	PEDOT:PSS	PPy(PF_6)	AN	10k	EMImOTf	AN	[-1,0.3]	600	0.56%	0.47%	-0.150	0.144	4%	18%	17%
	a	PEDOT:PSS	PPy(TFSI)	AN	10k	EMImOTf	AN	[-1,0.3]	600	0.11%	0.08%	-0.044	0.045	-3%	29%	26%
	b	PEDOT:PSS	PPy(TFSI)	AN	10k	EMImOTf	AN	[-1,0.3]	600	0.10%	0.12%	-0.040	0.039	4%	37%	29%

	c	PEDOT:PSS	PPy(TFSI)	AN	10k	EMImOTf	AN	[-1,0.3]	600	0.24%	0.24%	-0.068	0.069	-1%	8%	6%
Effect of the electropolymerization and actuation solvent	a	PEDOT:PSS	PPy(TFSI)	AQ	10k	EMImOTf	AQ	[-1,0.3]	600	0.41%	0.40%	-0.068	0.065	5%	4%	3%
	b	PEDOT:PSS	PPy(TFSI)	AQ	10k	EMImOTf	AQ	[-1,0.3]	600	0.25%	0.26%	-0.046	0.045	2%	-3%	0%
	c	PEDOT:PSS	PPy(TFSI)	AQ	10k	EMImOTf	AQ	[-1,0.3]	600	0.86%	0.86%	-0.130	0.117	11%	15%	13%
	a	PEDOT:PSS	PPy(TFSI)	AQ	10k	EMImOTf	AN	[-1,0.3]	600	0.05%	0.03%	-0.040	0.039	2%	27%	24%
	b	PEDOT:PSS	PPy(TFSI)	AQ	10k	EMImOTf	AN	[-1,0.3]	600	0.20%	0.16%	-0.071	0.068	5%	18%	16%
	c	PEDOT:PSS	PPy(TFSI)	AQ	10k	EMImOTf	AN	[-1,0.3]	600	0.07%	0.06%	-0.032	0.031	6%	36%	29%
	a	PEDOT:PSS	PPy(TFSI)	AN	10k	EMImOTf	AQ	[-1,0.3]	600	0.77%	0.73%	-0.058	0.050	15%	-22%	-24%
	b	PEDOT:PSS	PPy(TFSI)	AN	10k	EMImOTf	AQ	[-1,0.3]	600	0.71%	0.62%	-0.132	0.122	8%	-22%	-40%
	c	PEDOT:PSS	PPy(TFSI)	AN	10k	EMImOTf	AQ	[-1,0.3]	600	1.36%	1.25%	-0.141	0.132	7%	2%	6%
Effect of thickness of PPy	a	PEDOT:PSS	PPy(ClO_4)	AQ	10k	$LiClO_4$	AQ	[-1,0.3]	600	0.20%	0.23%	-0.089	0.078	14%	-3%	-6%
	b	PEDOT:PSS	PPy(ClO_4)	AQ	10k	$LiClO_4$	AQ	[-1,0.3]	600	0.06%	0.23%	-0.145	0.128	14%	2%	1%
	c	PEDOT:PSS	PPy(ClO_4)	AQ	10k	$LiClO_4$	AQ	[-1,0.3]	600	0.21%	0.15%	-0.066	0.048	36%	35%	28%
	a	PEDOT:PSS	PPy(ClO_4)	AQ	20k	$LiClO_4$	AQ	[-1,0.3]	600	0.12%	0.11%	-0.077	0.066	16%	13%	10%
	b	PEDOT:PSS	PPy(ClO_4)	AQ	20k	$LiClO_4$	AQ	[-1,0.3]	600	0.38%	0.35%	-0.197	0.172	15%	16%	16%
	c	PEDOT:PSS	PPy(ClO_4)	AQ	20k	$LiClO_4$	AQ	[-1,0.3]	600	0.15%	0.15%	-0.086	0.066	30%	-28%	-68%
	a	PEDOT:PSS	PPy(ClO_4)	AQ	30k	$LiClO_4$	AQ	[-1,0.3]	600	0.12%	0.16%	-0.090	0.079	14%	14%	4%
	b	PEDOT:PSS	PPy(ClO_4)	AQ	30k	$LiClO_4$	AQ	[-1,0.3]	600	0.05%	0.09%	-0.145	0.128	14%	22%	12%
	c	PEDOT:PSS	PPy(ClO_4)	AQ	30k	$LiClO_4$	AQ	[-1,0.3]	600	0.09%	0.11%	-0.066	0.048	36%	8%	-2%
Effect of PEDOT dopant	a	PEDOT:Tos	PPy(ClO_4)	AQ	10k	$LiClO_4$	AQ	[-1,0.3]	600	0.07%	0.10%	-0.051	0.037	37%	1%	13%
	b	PEDOT:Tos	PPy(ClO_4)	AQ	10k	$LiClO_4$	AQ	[-1,0.3]	600	0.09%	0.09%	-0.037	0.031	19%	20%	9%

	c	PEDOT:Tos	PPy(ClO_4)	AQ	10k	$LiClO_4$	AQ	[-1,0.3]	600	0.00%	0.03%	-0.027	0.021	27%	9%	14%
PPyDBS-yarn in $LiClO_4$ Aq solution	a	PEDOT:PSS	PPy(DBS)	AQ	10k	$LiClO_4$	AQ	[-1,0.3]	600	3.31%	3.23%	-0.260	0.247	5%	-2%	1%
	b	PEDOT:PSS	PPy(DBS)	AQ	10k	$LiClO_4$	AQ	[-1,0.3]	600	0.90%	0.92%	-0.130	0.110	18%	-36%	-50%
	c	PEDOT:PSS	PPy(DBS)	AQ	10k	$LiClO_4$	AQ	[-1,0.3]	600	1.56%	1.52%	-0.183	0.167	10%	31%	5%
Dual Actuation	a	PEDOT:PSS	PPy(DBS)	AQ	10k	$LiClO_4$	AQ	[-1,1]	600	0.38%	0.37%	-0.066	0.065	2%	-26%	-18%
	a	PEDOT:Tos	PPy(ClO_4)	AQ	10k	$LiClO_4$	AQ	[-1,1]	600							
	b	PEDOT:PSS	PPy(DBS)	AQ	10k	$LiClO_4$	AQ	[-1,1]	600	0.26%	0.25%	-0.070	0.072	-3%	5%	12%
	b	PEDOT:Tos	PPy(ClO_4)	AQ	10k	$LiClO_4$	AQ	[-1,1]	600							
	c	PEDOT:PSS	PPy(DBS)	AQ	10k	$LiClO_4$	AQ	[-1,1]	600	0.35%	0.34%	-0.108	0.093	16%	-53%	-38%
	c	PEDOT:Tos	PPy(ClO_4)	AQ	10k	$LiClO_4$	AQ	[-1,1]	600							

Table S1: The reduction and oxidation strain and charge, as well as the Irreversible charge, in percentage, and the degradation during the oxidation and the reduction, also in percentage, of all experiments presented in the paper. The irreversible charge is calculated according to the formula: Irreversible charge = 100(Qred-Qox)/Qox, and the degradation values are calculated according to the formulas: Degradation red=100*(Qred(3rd cycle) - Qred(last cycle))/Qred(3rd cycle), Degradation ox= 100*(Qox(3rd cycle) - Qox(last cycle))/ Qox (3rd cycle), where red is reduction, ox means oxidation, and Q is the charge in C.*
